# Temporal variability in quantitative human gut microbiome profiles and implications for clinical research

**DOI:** 10.1038/s41467-021-27098-7

**Published:** 2021-11-18

**Authors:** Doris Vandeputte, Lindsey De Commer, Raul Y. Tito, Gunter Kathagen, João Sabino, Séverine Vermeire, Karoline Faust, Jeroen Raes

**Affiliations:** 1 KU Leuven – University of Leuven, Department of Microbiology, Immunology and Transplantation, Rega Institute, Herestraat 49, B-3000 Leuven, Belgium; 2 VIB, Center for Microbiology, Kasteelpark Arenberg 31, B-3000 Leuven, Belgium; 3 Translational Research Center for Gastrointestinal Disorders (TARGID), KU Leuven B-3000 Leuven, Belgium

**Keywords:** Symbiosis, Microbiome, Microbial ecology

## Abstract

While clinical gut microbiota research is ever-expanding, extending reference knowledge of healthy between- and within-subject gut microbiota variation and its drivers remains essential; in particular, temporal variability is under-explored, and a comparison with cross-sectional variation is missing. Here, we perform daily quantitative microbiome profiling on 713 fecal samples from 20 Belgian women over six weeks, combined with extensive anthropometric measurements, blood panels, dietary data, and stool characteristics. We show substantial temporal variation for most major gut genera; we find that for 78% of microbial genera, day-to-day absolute abundance variation is substantially larger within than between individuals, with up to 100-fold shifts over the study period. Diversity, and especially evenness indicators also fluctuate substantially. Relative abundance profiles show similar but less pronounced temporal variation. Stool moisture, and to a lesser extent diet, are the only significant host covariates of temporal microbiota variation, while menstrual cycle parameters did not show significant effects. We find that the dysbiotic Bact2 enterotype shows increased between- and within-subject compositional variability. Our results suggest that to increase diagnostic as well as target discovery power, studies could adopt a repeated measurement design and/or focus analysis on community-wide microbiome descriptors and indices.

## Abstract (web summary)

Here, the authors report quantitative daily gut microbiome variation of individual gut bacterial abundances in healthy individuals, linked to changes in transit time and diet, highlighting the potential need for multiple samplings for microbiome target identification and the development and application of reliable microbiome diagnostics.

## Introduction

Fecal microbiota signals have been put forward as promising biomarkers for disease and clinical progression, and diagnostic applications have already been proposed for several conditions (e.g., Inflammatory Bowel Diseases (IBD) and colorectal cancer)^1–5^. Furthermore, recent cross-sectional results suggest that whole-microbiota typing, and notably the Bacteroides 2 (Bact2) enterotype, could be used for identifying (disease-associated) dysbiosis^4,6,7^. While several cross-sectional studies have provided a good understanding of the inter-individual variability in microbiota composition and its drivers^8–15^, longitudinal, intra-individual microbiota variation understudied. Furthermore, none of the few longitudinal studies available used absolute abundances, which, in contrast to relative approaches, can provide information on the extent and directionality of changes in taxa abundance, are more easily linked to other quantitative data, and do not introduce compositionality effects in correlation analysis.^7^ In addition, no knowledge exists on absolute (vs. relative) temporal microbiota abundance variation^16–21^. To address this, we present a densely sampled time series of quantitative, fecal bacterial microbiome profiles of healthy women living in an industrialized country. We show substantial temporal variation for most major gut genera, as well as diversity indicators for both relative and quantitative abundance profiles. Stool moisture, and to a lesser extent diet, are the only significant host covariates of temporal microbiota variation, while menstrual cycle parameters did not show significant effects. We further find that the dysbiotic Bact2 enterotype shows increased between- and within-subject compositional variability.

## Results

### 20 dense fecal microbial time series of Belgian women

We collected 713 fecal samples from 20 Belgian women (age [33 ± 8; 25:51], Body Mass Index (BMI) [22.6 ± 3.5; 18.6:33.5]), in a daily sampling effort covering six weeks, together with a rich panel of participant metadata, blood values, and fecal calprotectin (Supplementary Data S1), comprising among others daily carbohydrate, protein and fat intake, medication, and stool characteristics. Given the paucity of data on menstruation cycle-microbiome interactions yet indications of hormone effects^8^, data on menstrual cycle was collected and corresponding hormone levels were inferred. Both stool consistency (Bristol Stool Score (BSS) [3.5 ± 1.2; 1:7] and moisture content [73 ± 8%; 52%:93%]) of the fecal samples fell within normal ranges (Supplementary Data S1)^8^. No participant had major gastro-intestinal disease, took antibiotics in the three months before the study, or used hormonal contraceptives. A few deviations from healthy ranges were noted for the measured molecular markers and anthropometric measurements at the start and end of the study, yet all participants were in good health during the trial. An exception was participant 808 who had an intestinal infection. Daily recorded dietary data show participants mostly had a Western-style dietary pattern with on average 50% (28–62%), 34% (24–44%) and 15% (11–26%) of total energy intake (EI) coming from carbohydrates, fats, and proteins, respectively, and a daily intake of 13 g of fiber (7:30) (Supplementary Data S1, Supplementary Fig. 1)^22^. Intestinal inflammation markers outside of the expected range could be linked to NSAID intake or infection (Calprotectin [17 ± 74; 0:384], clinical reference value = 200 µg/g)^23^. Quantitative Microbiome Profiles (QMPs) were determined through combined 16 S sequencing and flow cytometry^7^. Study sample microbiomes include all four enterotypes^24^ and both richness and microbial load ranges are consistent with previous reports on the Belgian population (Supplementary Fig. 1)^7,8^. Sample quality control based on community-wide methods, comparing between same- and other-person sample dissimilarities^16,25^ in combination with person-specific clustering^26^ (see methods) revealed two sample swapping events. All analyses were performed on a corrected dataset in which those samples were relabeled to the indicated originals.

### High intra- versus inter-individual variation

First, we assessed variation in absolute abundance of specific microbial groups, as well as global ecosystem readouts covering overall diversity and/or richness (alpha diversity), between sample overall compositional variation (beta diversity), and large community state shifts (enterotypes). All analyses were carried out using amplicon sequence variants grouped at genus-level.

**Fig. 1:**
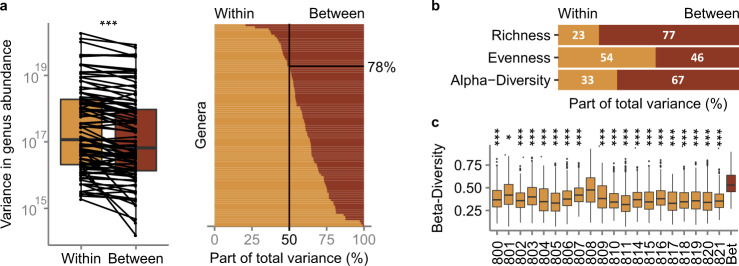
Extensive within- compared to between-subject variance in genus abundances are reflected partly in alpha-diversity but not in beta-diversity measures. **a** Within- versus between-subject variance in absolute genus abundance (Wilcoxon test, two-sided, *n* = 96, *P* = 1.75 × 10^−7^) (left) and within-subject and between-subject variance as part of the total variance in genus abundance (right) for all genera over the abundance threshold. **b** Within- and between-subject variance as part of the total variance in alpha diversity measures, for observed richness, Pielou evenness, and Shannon diversity index based on QMPs. **c** Beta-diversity, as assessed through Bray Curtis dissimilarity, between samples of the same individual (800–821), and between the first sample of each individual (Bet) based on QMPs. Significance of the differences within and between individuals were tested through an ANOVA on multivariate homogeneity of group dispersions (*N* = 20, *n* = 694). The body of the box plots represents the first and third quartiles of the distribution, and the median line. Whiskers extend from the quartiles to the last data point within 1.5×IQR, with outliers beyond. Significance levels: ***: 0.001, **: 0.01, *: 0.05. The same figure based on RMPs can be found in Supplementary Fig. 2.

**Fig. 2:**
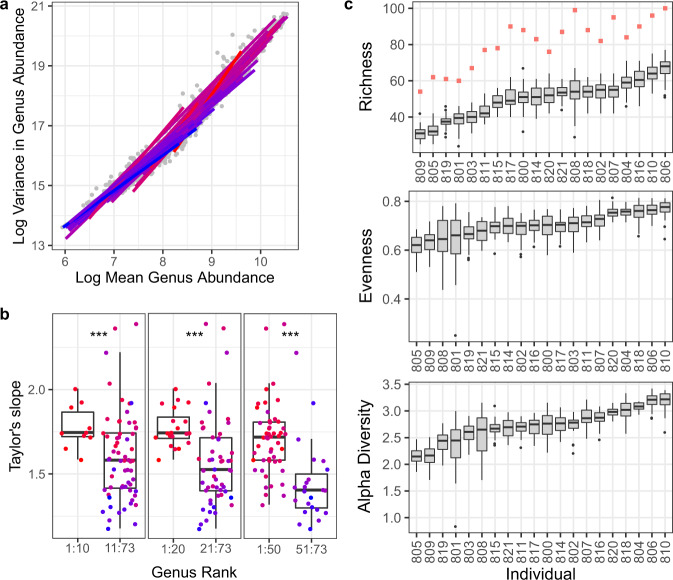
High-ranked genera are more stable with increasing mean abundance than low-ranked genera. Mean-variance relationships can be characterized by a linear relationship known as Taylor’s Law (TL). According to TL *log(variance)*_*g*_ = *b* *×* *log(mean)*_*g*_, where *b* is a species-specific constant and *g* indicates the group of measurements. For a given unit change in the log(mean) of population abundance, the TL’s slope equals the change in log(variance), which measures heterogeneity or scatter in the distribution of population abundance. A greater slope in the temporal TL means a greater degree of change in the temporal variance of genus population abundance with respect to its temporal mean. Genera for which the TL slope is 2 have about the same coefficient of variation in all subjects. Genera for which the TL slope is less than 2 show more temporal variation in subjects in which they are more abundant. The reverse for genera for which the TL slope is greater than 2. Here, we show Taylor slopes of high ranked genera (over all individuals) are generally higher, indicating more variation with increasing mean abundance, compared to low ranked genera. **a** Mean-variance relationships for all genera satisfying the abundance and prevalence criteria (genera with abundance >0.5%, present in >5 individuals in >3 samples). Regression lines are colored according to Taylor’s slope from less steep (blue) to steep (red). **b** TL slope of high versus low-ranked genera, for the top 10, 20, and 50 genera (*t*-test between TL slopes of top 10, 20, and 50 versus lower-ranked genera, two-sided, *P* = 0.00318, *P* = 0.0000285, *P* = 0.000188, respectively, *N* = 73). **c** Intra-individual variation in alpha diversity measurements, (top) observed richness with an indication of the total number of genera detected over all samples (squares), (middle) Pielou’s evenness index ‘J’, and (below) Shannon alpha diversity index for each individual (ID-number 800–821) (*N* = 20, *n* = 694). The body of the box plots represents the first and third quartiles of the distribution, and the median line. Whiskers extend from the quartiles to the last data point within 1.5×IQR, with outliers beyond. Significance levels: ***: 0.001, **: 0.01, *: 0.05.

We show extensive temporal variation in genus abundance (coefficient of variation in genus abundance [1.0 ± 0.7; 0.6:4.0], *N* = 73, genera with abundance>0.5%, present in >5 individuals in >3 samples, QMP, Supplementary Fig. 2). Interestingly, *Prevotella* abundances, known to vary greatly between individuals, also show remarkable within-subject variation. Using Intra Class Correlation coefficients (ICC) on genus abundances stratifying for individual, we assess the proportion of the within- and between-subject variance^27^. We show more than 78% of the genera varied more within than between persons over the study period (genera with abundance >0.5%, ICC < 0.5, Fig. 1, Supplementary Data S2). We noted large day-to-day abundance shifts: 72% of all genera show over 10-fold abundance shifts between consecutive samples (genera with abundance >0.5%, consecutive samples). In a few weeks, even more extensive changes in abundance can take place: 100-fold changes are no exception (100-fold changes were detected for 40% of the genera with abundance >0.5%, *N* = 20, *n* = 694, Supplementary Data S2). Shifts in taxonomic ranks are frequent and extensive: genera cover on average 19 [11–29] ranks over the study period (Supplementary Data S2). This said, most genera fluctuate, albeit strongly, around an equilibrium level (>97 ± 7.5% [72:100%] of all genera of a person with abundance >0.5%, Augmented Dicky Fuller test, two-sided, *P* < 0.05, *N* = 20, *n* = 694). All genera show a power-law relationship between variance and mean abundance over time (Taylor’s law^28^; *Q* < 0.001, adjusted *R*^2^ > 0.75 [0.92 ± 0.05], genera with abundance >0.5%, present in >5 individuals in >3 samples, Table 1). Most genera are more stable in subjects in which they are more abundant, yet some show an inverse relation (e.g., *Parabacteroides, Paraprevotella*, and *Methanobrevibacter*). In general, high-ranked genera are more stable with increasing mean abundance than low-ranked genera (*t*-test of Taylor slopes between high and low ranked genera, two-sided, *P* < 0.05 from rank 5 until 69, *N* = 73, Fig. 2).

Alpha-diversity, capturing both the number of taxa detected within a sample (richness) and their abundance distribution (evenness), varies substantially over different samples of the same person. In this cohort, 33% of the total variation in the Shannon diversity index can be attributed to temporal variation (ICC: 0.67). Richness shows less temporal variation (ICC: 0.77), but evenness varies even more within than between persons (ICC: 0.46, genera with abundance >0.5%, *N* = 20, *n* = 694, Fig. 1, Supplementary Data S2). Relative abundance variation shows a similar but less pronounced picture. Genus abundances vary substantially in time (Coefficient of variation [0.8 ± 0.6; 0.3:3.5], genera), *N* = 73, genera with abundance>0.5%, present in >5 individuals in >3 samples) but within- versus between-subject variation in genus abundance is less using relative data (36% of all genera note an ICC below 0.5 using RMPs—compared to 78% with QMPs). For RMPs, ICC values for richness, evenness, and alpha diversity, are 0.85, 0.46, and 0.64, respectively (Supplementary Fig. 3).

### Implications of high intra-individual variation in genus abundances for clinical research

As a whole, these results show that genus abundances show substantial day-to-day variation around an equilibrium state. Compared to RMPs, longitudinal QMPs show even more temporal variation. Indeed, absolute numbers also reflect the substantial day-to-day fluctuations in biomass, aggravating their temporal variance^7^. While previous reports (based on RMPs) stressed the equilibrium properties of the human gut microbiome, we here draw attention to its variability. Cross-sectionally determined biomarkers and targets are based on average differences between patients and controls (lumping together within and between person variability). Our results suggest that a single measurement does not estimate a person’s temporal average well, both using relative and quantitative profiling. Consequently, single-time-point diagnostics have a high risk of misclassification, as differences between patients and controls might fall within normal temporal variation, and target discovery suffers from high noise. Table 1 therefore provides the coefficient of variation in genus abundance for both RMP and QMP, which can serve as a reference for variation under normal conditions. The use of summary measures is recommended in cases where within-subject variation is high compared with between-group differences.

**Fig. 3:**
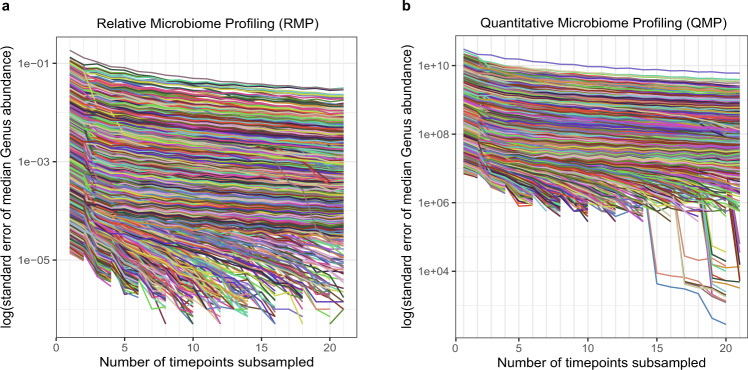
Gains in accuracy in the estimation of the equilibrium abundance are highest for the first five samples for both RMP and QMP. The availability of multiple timepoints allows improved estimation of the equilibrium genus abundance through summary measures (e.g., median). However, gains in accuracy decrease with additional timepoints. We calculated the error on the median genus abundances (*y*-axis) depending on the number of timepoints (100 most abundant genera, all participants, timepoints randomly chosen out of the full time series, 10.000 iterations). The elbow of the curve, a point that signifies an optimum in the trade-off between accuracy and sampling effort, lies roughly around 5 samples for both RMP (**a**) and QMP (**b**). Gains in accuracy level off afterwards as evidenced by the flatter curves.

Reducing abundance variation by averaging multiple timepoints is also expected to improve the identification of cross-sectional correlations. Poyet et al. showed that calculating median abundances over several timepoints enhanced the detection rate of cross-sectional taxon-taxon correlations in RMPs (10 longitudinal RMPs, 32 samples).^19^ To get a better idea of the improvements in the estimation of equilibrium abundances through increased sampling, we predicted the variance in our estimate of genus median abundance for a given sample size by randomly subsampling the time series at different levels of temporal resolution for both QMPs and RMPs (20 people, 21 samples, Fig. 3). As expected, we find that the variance in our estimate is reduced by collecting more time points and most is gained in the first additional samples. Similar to Poyet et al., the optimum in the trade-off between accuracy and sampling effort, lies around 5 samples for both RMP and QMP. However, the cut-off value is less clear, meaning that improvements in accuracy are more equally divided over the samples. In addition, although the recommendation by Poyet et al. (sampling 5 or more samples) will yield improved accuracy, it does not take into account the substantial financial and operational cost of increased sampling. To introduce temporal replicates while limiting expenses, we feel that collecting minimally three longitudinal samples allows calculating equilibrium abundances with substantially higher accuracy, as well as estimating temporal variation with a minimum number of samples. Depending on the study objective, size, design, and population, the trade-off between additional information and increased longitudinal sampling will differ and adjustments should be made accordingly. For now, these decisions would be mostly guided by general statistics and first insights into temporal microbiome variation, yet a wider adoption of a longitudinal sampling design will extend our ability to make practical recommendations.

Interestingly, the lower temporal variance of RMP versus QMP might make this kind of profiling, although further from reality, potentially more suited for diagnostic biomarker identification, especially if only single-time point measurements are available. Indeed, the reduction of information going from absolute to relative numbers would work to its advantage. However, when identifying clinical targets and understanding pathomechanisms, only absolute abundances are the relevant measure, as they determine the total biomass and production capacity of transcripts, proteins, and other compounds. All associations with host factors, ranging from immune markers to metabolomics (which are all expressed in absolute numbers), and ecological interpretations would benefit from having the absolute instead of relative numbers^7^.

We next assessed the temporal and cross-sectional core genera within our dataset^8,17,29^. Based on all samples within a QMP time series, about 39 ± 7% [16:50%] of a person’s genera are detected in more than 95% of the samples (=Temporal core genera; genera with an abundance>0.5%, *N* = 20, *n* = 694, Table 1, Supplementary Fig. 4, Supplementary Data S3). Temporal core size [30 ± 7; 16:42 genera] falls within 90% of the estimate based on the total time series from 11 samples onwards for all non-perturbed time series (Supplementary Fig. 4). Genera shared between all individuals (=cross-sectional core genera) constitute 10% of all detected genera in this dataset (17/176, genera with an abundance>0.5%, *N* = 20, *n* = 694, Supplementary Data S3). Nine taxa belong to both the cross-sectional and temporal core, namely *Alistipes*, *Bacteroides*, *Blautia*, *Clostridium cluster XIVa, Faecalibacterium*, *Roseburia*, *Ruminococcus*, and two groups of unclassified sequence variants within the families Lachnospiraceae and Ruminococcaceae. About three quarters of the individuals (14/20) carried between one and four person-specific genera, representing about 16% of all detected genera (28/176). Some of these person-specific genera are only detected once (11/28), but most are present in several samples and some even belong to the temporal core of an individual (3/28).

**Fig. 4:**
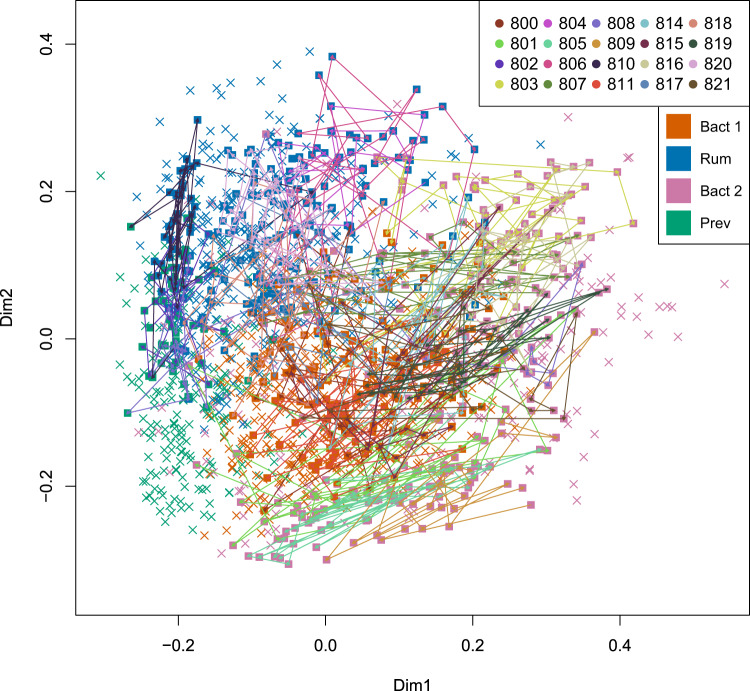
Visualization of microbiome composition trajectories. Principal Coordinate Analyses of RMPs of the study cohort samples (squares) as well as 1104 FGFP participants (crosses), colored according to enterotype, namely Ruminoccocaceae (Rum, blue), Bacteroides 1 (Bact1, orange), Bacteroides 2 (Bact2, Pink), Prevotella (Prev, green). Arrows indicate the sampling sequence in time, color coded for participant.

Comparison of intra- and inter-individual variability in beta-diversity, or the dissimilarity between samples, shows day-to-day variation is more pronounced than expected based on previous research.^16,19,29–31^ Yet, as expected, whole-community dissimilarity remains generally larger between than within individuals (ANOVA on multivariate homogeneity of group dispersions on BC dissimilarities between individuals (first sample) and within each individual (full time-series), two-sided, *N* = 20, *Q* = 0.33 (participant 808) and *Q* < 0.05 (all other participants), Fig. 1c, Supplementary Data S4) but at several occasions, equally large shifts can be noted within as between persons. Notably, such large day-to-day shifts (illustrated using PCoA; Fig. 4) mostly occurred in the absence of major perturbations (with the exception of one infection event).

**Fig. 5:**
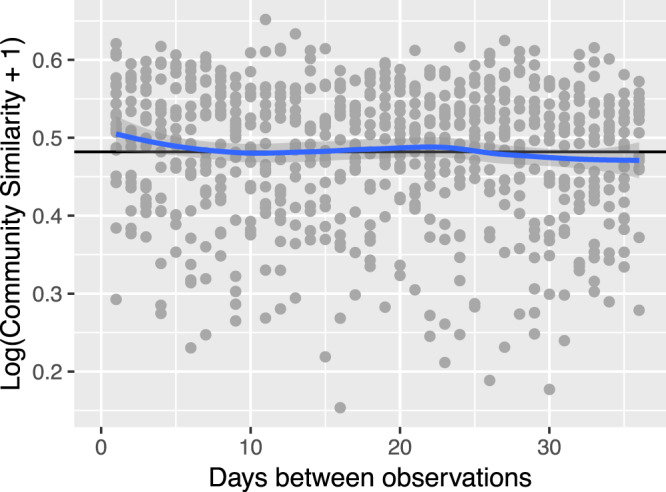
Longitudinal sampling can be done over the course of several weeks in normal situations without perturbations. There is no similarity decay in microbiota composition over 4 weeks. Linear model of log-transformed similarities (= 1 - community dissimilarities (BC)) between time points of interpolated QMP time series against the number of days elapsed (*R*^2^ = 0.013, *P* > 0.05). Data points of each day were randomly subsampled to the lowest amount of observations for a day. Length of the evaluated time span was limited to include data of all 20 participants at each value (1–36 days).

Assessing similarity between samples over increasing time spans^32^, we show time does not have an incremental effect on gut microbiota composition over 36 days (non-significant Similarity Decay Analysis, *N* = 19, *n* = 3780, *R*^2^ = −0.00028, *P* = 0.84, Fig. 5, Supplementary Data S4). This means that fecal microbial communities differ as much from baseline after a day, as they do after one week or one month. Consequently, these results suggest that it does not matter at what time interval one takes multiple samples to estimate variation or calculate summary measures, at least for the 36-day time interval tested here.

Next, we assessed community variation with the concept of enterotypes, using the Dirichlet Multinomial Mixtures approach^33^, which classifies samples in categories based on similarities between gut microbiota composition in different individuals. Previous cross-sectional population studies identified four such states in healthy individuals^8,24,26,34,35^. One of these, the Bact2 enterotype has been shown to be more prevalent among people with Crohn’s disease, ulcerative colitis, primary sclerosing cholangitis, depression, obesity, and certain multiple sclerosis subtypes^4,6,36,37^. These states were previously shown to be relatively stable over longer time intervals: 84% of the people did not change enterotypes in an analysis using two single timepoints as collected during the Human Microbiome Project (sampling interval between 30 and 451 days, median 224 days, *N* = 100)^24,34^, but their daily variation remains unassessed. Here we enterotyped each sample on the background of >1000 samples from the Flemish Gut Flora Project (FGPF) to include population-level information^8^. Individual trajectories all show movement around one center supporting the interpretation of microbiota being in a stable state^38^ (Fig. 4). Yet, transitory enterotype switches, which represent some of the major compositional changes within a person (BC dissimilarity between same- vs. different-enterotyped consecutive samples, two-sided Wilcoxon test subsampled to the same number of observations in both groups, *n* = 123, *P* = 0.028, Supplementary Data S4), do occur occasionally. We next asked whether the observed enterotype transitions were in accordance with the extent of the compositional shifts needed to bring them about. In other words, are transitions that require large compositional shifts also less frequent (and vice versa)? We studied the discrepancies between expected transition rates, based on enterotype prevalence, and observed, and contrasted these with the compositional dissimilarity within or between enterotypes (Fig. 2, Supplementary Data S4). For each transition, within or between enterotypes, we calculated the expected probability of an A–B transition as the number of A–B transitions possible within the dataset divided by the total number possible transitions within the dataset, and compared it with the observed number of enterotype transitions.

**Fig. 6:**
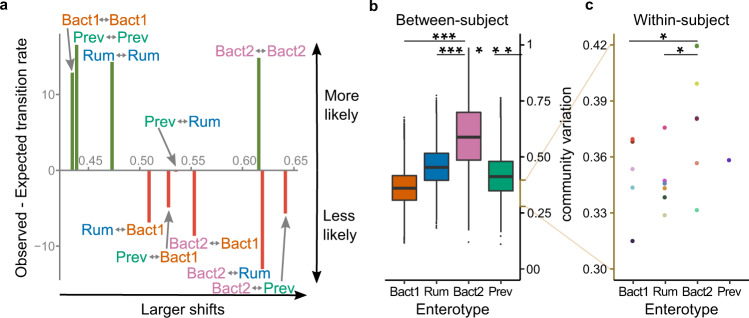
Bact2 enterotype assignment is as stable over time as other enterotypes despite higher between- and within- subject community variation. **a** For all enterotype switches, discrepancies between expected and observed switching rates (standardized residuals of a Chi Square goodness of fit test with 10 categories for all possible enterotype switches, *P* = 2 × 10^−16^) (*y*-axis), were set out against community variation (median BC dissimilarity of the constellation using all applicable samples in the study cohort, *N* = 20, *n* = 694) (*x*-axis). **b** Between-subject community variation per enterotype, calculated as the BC dissimilarity between samples of different individuals of the same enterotype within the FGFP cohort (*N* = 1103; *N* = 401,331,186,185 for the Bacteroides1 (B_1_), Ruminococcaceae (R), Bacteroides2 (B_2_), and Prevotella (P) enterotypes, respectively). Between-subject community variation is significantly higher in Bact2 versus all other enterotypes (ANOVA on multivariate homogeneity of group dispersions on BC dissimilarities between individuals across enterotypes, *N* = 1103, *Q* < 10^−29^ for all comparisons with Bact2). **c** Within-subject community variation per enterotype, calculated as the median BC dissimilarity between all samples of an individual enterotyped as Bact1, Rum, Bact2, or Prev for the majority of their samples (*N* = 19, excluding participant 808). Within-subject community variation is significantly higher in Bact2 versus Bact1 and Rum enterotypes (Dunn test, two-sided, *N* = 19, *P* = 0.0392, and *P* = 0.0096, respectively). The body of the box plots represents the first and third quartiles of the distribution, and the median line. Whiskers extend from the quartiles to the last data point within 1.5×IQR, with outliers beyond. Significance levels: ***: 0.001, **: 0.01, *: 0.05.

As expected, within enterotype transitions are more frequent than one would predict based on a random process, hence confirming enterotype stability (Chi square test goodness of fit with 10 categories for all possible enterotype switches, *P* = 2 × 10^−16^, Fig. 6a, *y*-axis, Supplementary Data S4). We next contrasted switches with the median compositional dissimilarity. As expected, for Bact1, Prev, and Rum enterotypes, within-enterotype transitions are linked with the least compositional difference between corresponding samples. However, the Bact2 enterotype shows a distinct pattern: it is as stable as the other enterotypes (the difference between the observed and expected transition rate of Bact2–Bact2 transitions is similar to those of other same-enterotype transitions), yet its compositional variability is much larger and even exceeds that of inter-enterotype constellations (Dunn test, BC dissimilarity of Bact2–Bact2 versus Rum–Bact1, Prev–Bact1, Prev–Rum, Bact2–Bact1 constellations, *N* = 20, *P* < 10^−5^ for all mentioned comparisons, Fig. 6a). Compositional variability within the Bact2 enterotype as tested here is the result of both compositional dissimilarity between and within persons. We therefore also quantified both components separately, using cross-sectional data of the Flemish Gut Flora Project (FGFP) and this studies longitudinal data to estimate between- and within-subject variability, respectively. The population-level data of the FGFP shows compositional dissimilarity across Bact2-enterotyped individuals is significantly higher than in all other enterotypes (ANOVA on multivariate homogeneity of group dispersions on BC dissimilarities between individuals across enterotypes, *N* = 1103, *Q* < 10^−29^ for all comparisons with Bact2, Fig. 6b). Our longitudinal study data shows the same is true for within-subject variability: compositional variation in time is significantly higher for those individuals with the majority of their samples in the Bact2 enterotype than for those in the Bact1 and Rum enterotype (Dunn test of median BC dissimilarity within individuals across enterotypes, two-sided, *N* = 19, *P* = 0.0392 and *P* = 0.0096, respectively, Fig. 6c). This observation suggests a dynamic component to the so-called ‘Anna Karenina principle’, which states that dysbiotic communities (Bact2) tend to vary more strongly than non-dysbiotic communities, as was observed in cross-sectional sense in IBD patients^26,39,40^. Consequently, repeated measurements are likely even more important to estimate equilibrium abundances in disease cohorts. Finally, we find Prev–Rum switches to be more likely than all other inter-enterotype switches, even those between more similar constellations (Chi square test goodness of fit, *P* = 2 × 10^−16^, Fig. 6a, *y*-axis, Supplementary Data S4).

Next, we assessed the reasons behind the observed patterns of temporal variation and determined to what extent differences in host-associated variables can explain microbiota shifts. To link the dietary data and microbiome profiles for each individual we calculated a personal lag time, adjusting a standard 24 h lag with information on cross-correlation of the dietary data with moisture content (see methods). Next to dietary data, such as protein, carbohydrate, fat, and fiber intake, we included data on medication, energy expenditure, time of sampling, estimated levels of ovarian hormones, and stool moisture content. Despite considerable intra-personal variation, none of these factors show significant effects, except for stool moisture content and, to a lesser extent, dietary parameters. We here show stool moisture-associated microbiota changes observed within individuals are qualitatively similar to those between individuals, suggesting the observed intra-individual variation might partly be responsible for the reported inter-individual differences.

**Fig. 7:**
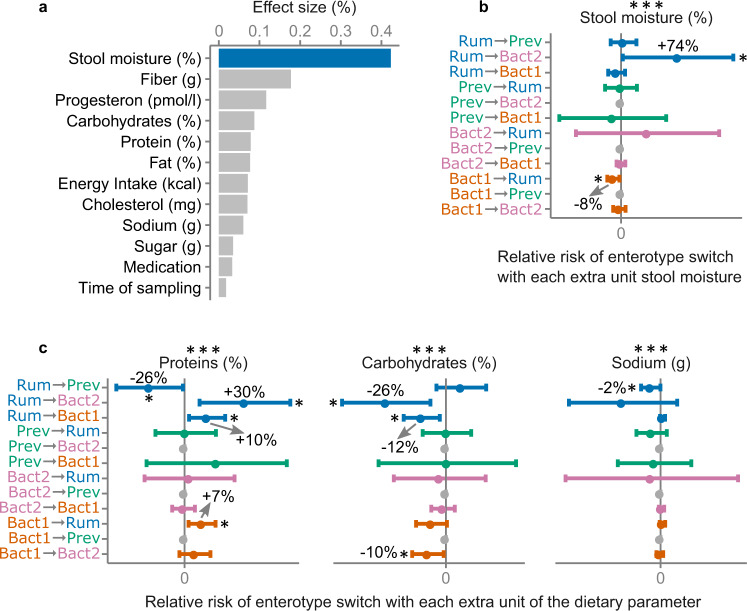
The only host covariates of temporal microbiota variation reaching significance in current cohort are transit time, as assessed through stool moisture, and diet. **a** Independent effect sizes (adjusted R2) of non-redundant covariates on microbiome community variation corrected for individual effects (dbRDA on a BC dissimilarity matrix of QMPs, *N* = 19, *n* = 661). The only variable significantly contributing to microbiome community variation in a combined model corrected for individual effects was stool moisture (blue, stepwise dbRDA on a BC dissimilarity matrix of QMPs, *N* = 19, *n* = 661, *P* = 0.002, gray, variables not entering the stepwise dbRDA model). **b** The relative risk of an enterotype switch with each increase in stool moisture (*x*-axis), set out for each possible enterotype switch (*y*-axis), reveals some of the observed Rum-enterotype switches can be related to changes in passage rate (*N* = 20, *n* = 709, *P* < 0.05, similar results for BSS score, Supplementary Fig. 4). Switches are color coded according to data availability: no data (gray), and subsequently to direction of the switch: away from the Ruminococcaceae (R, blue), Prevotella (P, green), Bacteroides2 (B_2_, pink), and Bacteroides1 (B_1_, orange) enterotype. Error bardots indicate the median value and whiskers extend to the 95% confidence interval. **c** The relative risk of an enterotype switch with each increase of the dietary parameter (*x*-axis), set out for each possible enterotype switch (*y*-axis), with graphical parameters similar to b (*N* = 20, *n* = 709). Summary statistics on carbohydrate, protein, and sodium intake can be found in Supplementary Table S1. Significance levels: ***: 0.001, **: 0.01, *: 0.05.

Assessing explanatory power of all variables with a stepwise distance-based redundancy analysis (dbRDA) shows the only factor significantly explaining total variation in gut microbiota composition beyond individual (50%), yet to a limited extent, is stool moisture content (0.4%, forward stepwise dbRDA on a BC dissimilarity matrix of QMPs, *N* = 19, *n* = 661, *P* = 0.002, Fig. 7a). This high explanatory power of individual and low contribution of metadata variables is in line with results of population-level cross-sectional studies^8–15^.

We investigated possible causes of enterotype shifts by evaluating the collected metadata and community characteristics upon switches using a multi-state Markov model. Such models describe a process in which an individual moves through a series of states in time and allows time-dependent covariates to be fitted to transition intensities^41^. Covariates with independent significant effects included all dietary metadata and transit time-related parameters, as well as time of sampling and medication intake (Log likelihood ratio test, *Q* < 0.05, *N* = 19, *n* = 661, Supplementary Fig. 5). However, also here, only transit time and some dietary data could be linked to specific enterotype shifts (Fig. 7b, c). Given the Bact2 disease association, knowing which parameters induce shifts away from this enterotype would be of interest, yet none of the investigated parameters were significantly associated to such transitions. Previous cross-sectional studies showed that the differences between Prev/Bact and Rum enterotyped individuals are associated with differences in passage rates: Rum enterotypes are high richness, high load constellations with a high proteolytic/saccharolytic functional profile, which are more prevalent in individuals with hard stools and/or slow transit, while Prev/Bact enterotypes show the opposite trend^7,8,42,43^. These differences have been postulated to arise, at least in part, through time-dependent gut ecosystem maturation^44^. With time, microbial density and richness increase due to prolonged growth possibilities, water is re-absorbed—resulting in firmer stools—, and a functional shift from saccharolytic to proteolytic fermentation takes place upon the depletion of readily fermentable substrates^44,45^. Here, transit time-associated parameters confirm this postulated shift towards a Rum constellation with increased passage rates and vice versa: one-way Bact1-to-Rum shifts are 8% less likely with every percent increase in stool moisture, while one-way Rum-to-Bact2 shifts are 74% more likely (relative risk with each unit increase (risk ratio) for moisture content: 0.92 and 1.74, respectively, *P* < 0.05, based on a multi-state Markov model. Similar results for BSS score). Interestingly, softer stools did not associate with a movement towards a Prev constellation^8,43,46^. Consistent with the increased proteolytic/saccharolytic potential of the Rum enterotype compared with the Prev enterotype^47^, a high intake of proteins seems to prevent shifts from Rum to Prev constellations (risk ratio = 0.85, *P* < 0.05). High sodium intake, which might increase colonic water reabsorption and lead to lower stool moisture content^48^, seems to have a similar effect (risk ratio 0.19, *P* < 0.05). Other noteworthy associations between dietary parameters and enterotype shifts include the one-way Rum-to-Bact2 and Rum-to-Bact1 transitions, which are more likely to occur with high protein and low carbohydrate consumption (risk ratio = 1.30 and 0.84, 1.10, and 0.93, respectively, *P* < 0.05). At the same time, we observe that the inverse, one-way Bact1-to-Rum shifts are also significantly associated with higher protein consumption (risk ratio = 1.07), suggesting that Bact1-Rum switches, in any direction, are triggered by other factors under high-protein consumption, or that major dietary disturbances per se can cause switches between these two states.

We further evaluated the temporal effect of the metadata on microbiota community characteristics and specific genus abundances using (Generalized) Linear Mixed Models ((G)LMMs). Again, we discovered a temporal effect in addition to the between-subject effect, for the earlier reported negative correlations between richness and microbial load with stool moisture. We find a negative correlation between richness as well as microbial load with stool moisture over all persons including temporal variation (Linear Mixed Model (LMM) with varying intercept and slope, *N* = 20, *n* = 694, *P* = 0.012 and 1.99 × 10^−5^, Stand. Est. = −1.14 and −2.2 × 10^10^, respectively), while a between-person analysis on a cross-sectional subset of the study cohort failed to pick up the trend for richness but not microbial load (LMM, *N* = 20, *n* = 20, *t* = 1, *P* = 0.69 and 0.015, Stand. Est. = 0.85 and −3.3 × 10^10^, respectively, Supplementary Data S5). Within subjects, the same relationships hold for the majority of individuals (Spearman test, two-sided, rho<0 for 16/20 and 19/20 participants, *P* < 0.05 for 3/16 and 11/19 of those, for richness and microbial load respectively, Supplementary Data S5). Other metadata variables did not significantly correlate with richness or microbial load, except for the time of sampling (morning/noon/evening/night) which correlated positively with microbial load (LMM, *N* = 20, *n* = 694, *Q* = 0.0077, Stand. Est = 8.42 × 10^9^). This might indicate a circadian rhythm effect^49,50^, but more likely also reflects transit time differences.

Validation of previously discovered genus abundance—stool moisture associations in a population-level cross-sectional study^8^, confirmed 5/13 associations, namely decreased *Roseburia* abundance and increased abundance of *Oscillibacter*, *Akkermansia, Ruminococcus*, and a group of unclassified members of the Ruminococcaceae, with lower moisture contents (LMM, *Q* < 0.05) in this longitudinal data set, suggesting a within-subject effect in these correlations. Although gut microbes might affect moisture content, the reverse is much more likely. These results therefore further support the relative importance of transit time in shaping gut microbiota composition. In order to identify taxon-metadata associations beyond stool consistency effects, we used GLMMs correcting for individual as well as moisture content. This data-driven approach identified no significant correlations (Supplementary Data S5). Within-subject coefficients of variation in genus abundance were not associated with within-subject coefficients of variation nor median values of collected metadata (Pearson correlation, genera with abundance>0.5%, present in >5 individuals in >3 samples, *Q* > 0.05, *n* = 803 = 73 × 11, respectively, genera and metadata variables).

## Discussion

Our observations challenge the current prevailing idea of inter-personal variation dwarfing intra-personal variation in gut microbiomes^12,16,30,51–54^. The 20 densely sampled time series together with the previously gathered population level data, allowed observation of substantial variation in within- versus between-subject genus abundances. This has important clinical implications. First, the ability to detect differences between individuals are reduced when within-subject variability is large in comparison to between-subject variability^55,56^. Consequently, gut microbiome discovery cohorts require large sample sizes to detect effects, as has become clear in recent years^8,10^. To resolve power issues, much could be gained from the calculation of summary measures^57–59^. Second, we show that single measurements do not estimate the equilibrium abundance well, therefore, repeated measurements will often be needed for diagnostic purposes. Third, we here show that community descriptors/indices such as beta diversity, enterotypes or stable states (centroids) are more stable than individual markers, and therefore—when clinically relevant to the pathology at hand—might have better diagnostic properties. Furthermore, a dynamic Anna Karenina principle might apply to human gut microbiomes, in other words, increased within-subject variability in microbiome composition might be a hallmark of (disease-associated) dysbiosis^38^. Consequently, it might be clinically relevant to not only characterize enterotypes or stable states, but also the variation in gut microbiome composition over time itself. Our results show that substantial temporal variation is also present in relative abundance-based profiling, yet much more pronounced with absolute abundance profiling. This indicates that microbiome temporal variation has, until now, been gravely underestimated and emphasizes the importance of using QMP approaches for target identification in clinical microbiome analyses. However, if the sole purpose of the study is the diagnostics-oriented detection of differences between groups (e.g., healthy and disease), RMPs might be the better option because of the reduced temporal variation.

Except for stool moisture and some dietary parameters, effect sizes of the extensively collected other metadata variables on gut microbiota composition over time was limited. The absence of general dynamic microbiota-metadata associations within this cohort are in line with a recent longitudinal study of RMPs, which observed highly personal dynamic profiles in which some diet-microbiome associations had opposite signals depending on the individual^60^. Although this could be partly due to the size (*N* = 20, *n* = 713) and characteristics of the study cohort, as well as data collection (e.g., dietary data records are only partly trustworthy^61^, and conventional nutrient profiles are less informative than food choices^60^), it might also suggest highly individual responses or the influence of additional, currently unknown covariates and/or intrinsic dynamics^38,62^. It seems likely that intrinsic factors like interactions between community members, growth rates, or immigration outweigh the effects of most of the currently explored metadata variables^62,63^.

Furthermore, the dynamics of microbial community composition trajectories in ordination plots suggests the existence of stable states, which is a prerequisite for the interpretation of enterotypes as alternative stable states in a multi-stable system^38,62^. However, while normal day-to-day events seem to be sufficient to trigger transient enterotype switches, we did not observe stable, long-term state transitions, in line with the idea that stronger perturbations are required to trigger a switch to an alternative stable state. That said, part of the observed temporal variation can be explained using the concept of ecosystem maturation with increased residence time^44^. Moreover, fluctuations in protein, carbohydrate/fiber, and sodium intake also seem to affect microbiome profiles over time. This is in line with results of other longitudinal and intervention studies, which report effects of (animal) protein^19,64^ as well as a range of different fiber sources^18,64–66^. We thus find that previously identified between-subject metadata-microbiota associations are also valid for longitudinal variation. These results again underscore the importance of including transit-time measurements/proxies as well as dietary information in clinical studies to disentangle microbiota-disease signals.

In conclusion, this study shows substantial temporal abundance variation of the gut microbiota around an equilibrium. Variation can be partly explained by moisture content and diet, yet a large part is unexplained and likely linked to intrinsic dynamics and ecology of the gut microbial ecosystem. Enterotypes differ in between- and within-subject compositional variability. These results are relevant for clinical study design, target identification, and diagnostics.

## Methods

### Ethical compliance

All experimental protocols were approved by the Commissie Medische Ethiek, UZ KU Leuven. Ethical approval of the study protocol was obtained (B322201525874). Study design complied with all relevant ethical regulations, aligning with the Declaration of Helsinki and in accordance with Belgian privacy.

### Study cohort

The study cohort consisted of twenty women recruited in the Flemish region near the university hospital (Leuven, Belgium) through a newsletter directed at FGFP participants as well as flyers distributed throughout the hospital. All participants gave their informed consent. Women were eligible to participate if they were aged between 16 and 55 years. Exclusion criteria were the use of any type of hormonal contraception three months prior to or during the study, the use of a copper intrauterine device, antibiotic treatment three months prior to study onset, pregnancy, the presence of inflammatory bowel disease or any type of bowel cancer. Volunteers received the provided smart phone as compensation for participation after completion of the study. Selection bias could have been induced through the recruitment channels (people interested in gut microbiome research tend to have gut problems or be related to people with gastrointestinal diseases), strict exclusion criteria, and smart phone use. Of twenty-two recruited volunteers, two did not complete the study protocol and were excluded from analyses.

### Sample and metadata collection

At enrollment and closure of the study medical staff recorded participants’ gender, age, height, and weight and took a blood sample (Supplementary Data S1). On the first day of menses following enrollment participants started collecting fecal samples at each defecation, with a maximum of one sample per day. They recorded sampling data such as stool consistency, time of sampling, and time between defecations, using a study smartphone. This device was further used to track physical activity, and record dietary intake through the application *MyFitnessPal*^67^ and menstrual cycle data through the application *Glow*^68^.

#### Stool moisture

Stool moisture content was determined on 0.2 g of frozen homogenized fecal material (−80 °C) as the percentage of mass loss after lyophilization.

#### Calprotectin

The Bühlmann^®^ Smart Prep Faecal Sample Preparation Kit and Bühlmann^®^ FCAL^TM^ ELISA kit were used for, respectively, FCal extraction and quantitative determination of human calprotectin from selected fecal samples. The test measures a calprotectin antigen by sandwich ELISA and was used according to the kit’s guidelines.

#### Hormones

Progesteron and estrogen levels were inferred by combining the information on the day of menstrual cycle with a standard profile over a 28-day menstrual cycle. The standard profile was based on measurements of endogenous progesteron and estrogen concentration throughout the menstrual cycle as collected for 300 cycles stemming from 12 studies, aligned by lutenizing hormone peak and including fourteen days prior and post lutenizing hormone peak (data shared by Jennie Lovett and Beverly Strassman)^69^. The standard profile was extrapolated or shortened to fit the cycle length of each individual in the study cohort. As the time between ovulation and menses (14 days) fluctuates a lot less than the time between menses and ovulation (10–21 days) we only added or substracted values at the beginning of the cycle in order to obtain the personal profile. Values beyond the standard 28-days were estimated by the value of day 1 increased by the value obtained by multiplying the difference between levels of day 1 and 28 with an exponentially decreasing function e^-*x*^, where *x* stands for each additional day beyond 28 days.

### Microbial load determination through flow cytometry

For cell counting, 0.2 g of frozen fecal aliquots were diluted 100,000 times in physiological solution (8.5 g/l NaCl; VWR International). In order to remove debris from the fecal solutions, samples were filtered using a sterile syringe filter (pore size 5 μm; Sartorius Stedim Biotech GmbH). Next, 1 ml of the microbial cell suspension obtained was stained with 1 μl SYBR Green I (1:100 dilution in dimethylsulfoxide; shaded 15 min incubation at 37 °C; 10,000 concentrate, Thermo Fisher Scientific). The flow cytometry analysis of the microbial cells present in the suspension was performed using a C6 Accuri flow cytometer (BD Biosciences), according to previously published methods^7^.

Fluorescence events were monitored using the FL1 533/30 nm and FL3 > 670 nm optical detectors. Forward and sideways-scattered light was also collected. The BD Accuri CFlow software was used to gate and separate the microbial fluorescence events on the FL1–FL3 density plot from the fecal sample background. A threshold value of 2000 was applied on the FL1 channel. The gated fluorescence events were evaluated on the forward–sideways density plot, to exclude remaining background events and to obtain an accurate microbial cell count. Instrument and gating settings were identical for all samples (fixed staining–gating strategy, Supplementary Fig. 8).

### Microbiota phylogenetic profiling of the study cohort

#### Sequencing data pre-processing

Fecal microbiome profiling of the study cohort was performed as described previously^8^. Briefly, DNA was extracted from about 200 mg fecal material using the MoBio PowerMicrobiome RNA isolation kit. Study samples were randomized and extraction blanks were included for every batch. The V4 region of the 16 S rRNA gene was amplified with primer pair 515 F/806 R (GTGYCAGCMGCCGCGGTAA/GGACTACNVGGGTWTCTAAT, respectively) modified to contain a barcode sequence between each primer and the Illumina adaptor sequences to produce dual-barcoded libraries^70^. Size selection, before Illumina sequencing, was performed using Agencourt AMPure to remove fragments below 200 bases. Sequencing was performed on the Illumina HiSeq 2500 platform (HiSeq-Rapid SBS kit v2 500 cycles, 2*250 PE) at the VIB Nucleomics core laboratory (Leuven, Belgium). After de-multiplexing with sdm as part of the LotuS pipeline^71^ without allowing for mismatches, fastq sequences were further analyzed per sample using the DADA2 pipeline (v. 1.6) rendering amplicon sequence variants^72^. In brief, after inspecting quality, sequences were trimmed to remove the primers and the first 10 bases after the primer, keeping only 200 bases and 130 for the R1 and R2 files, respectively. After merging paired sequences and removing chimeras, taxonomy was assigned using the formatted RDP training set ‘rdp_train_set_16’. The dataset contained 2393 amplicon sequence variants, which were grouped into 223 genus-level bins. Of these, 183 (84%) could be assigned to a known genus in the database (signified by a ‘g’ prefix in the taxon name), while the rest was named using the closest known taxonomic level (signified by a ‘uc’—for unclassified- prefix, followed by the taxonomic level: f–family, c–class, o–order, k–kingdom). Because of the subsampling procedures applied for relative and quantitative microbiome profiles, these contained 188 and 178 genus-level bins, respectively, of which 81% could be assigned at genus-level in both cases.

#### Relative microbiome profiles (RMPs)

For relative microbiome analyses, each sample was downsized to 10,000 reads by random selection of reads.

#### Quantitative microbiome profiles (QMPs)

For quantitative microbiome analyses, samples were downsized to even sampling depth, defined as the ratio between sample size (16 S rRNA gene copy number corrected sequencing depth) and microbial load (average total cell count per gram of frozen fecal material; Supplementary Data S1). 16 S rRNA gene copy numbers were retrieved from the ribosomal RNA operon copy number database rrnDB^73^. The copy number corrected sequencing depth of each sample was rarefied to the level necessary to equate the minimum observed sampling depth in the cohort, while assuring a minimum number of 500 reads in each sample and optimizing the chosen sampling depth to exclude as few samples as possible. In case of no copy number correction, this minimum threshold would represent about 2000 reads (average copy number of 3.88). A total of 13 samples were excluded for further analysis, 5 because of upfront exclusion due to low sequencing read numbers and 8 because they did not make the subsampling threshold. A QMP script to rarefy to an optimum even sampling depth is available on https://github.com/raeslab/temporal_variability_microbiome_qmp.

### Quality control & technical variation

All samples were sequenced in 4 different runs. Each run included a positive control (a fecal sample processed as all other samples), negative control (only reagents), and mastermix (PCR reagents). Negative controls only had a few reads (1–58, median: 23) for in total 15 genera (Supplementary Data S1). Reads belonging to chloroplasts and mitochondria were removed from the RMPs and QMPs before analyses. Technical variation was determined based on the RMPs of positive control samples (1-3/run) at 10,000 reads (Supplementary Data S1). Positive control samples had similar abundance profiles and clustered tightly together in a PCoA together with the RMPs of >1000 Flemish individuals. BC dissimilarity between runs was not larger than within runs (Supplementary Fig. 5). The variation observed within technical replicates was several times lower than the variation observed within individuals, for Shannon diversity index (standard deviation (sd): 0.09 versus median sd: 0.17) and Pieloux evenness (sd: 0.01 versus median sd: 0.04), but not richness (sd: 6, versus median sd: 5). The variable richness measurements within the technical replicates are not due to the subsampling process (genus richness could be considered stable from 5000 reads onwards and controls were subsampled to 10,000 reads) but to inconsistent detection of very low-abundant genera (<0.32%, Supplementary Fig. 6). To assess variation in taxon abundance or presence, we therefore applied an abundance threshold of 0.5%.

### Statistical analyses

All analyses were performed in *R*. All statistical tests used were two-sided, unless specified otherwise. Microbiome data was analyzed using amplicon sequence variants grouped at genus-level. Multiple testing was performed with the Benjamini–Hochberg procedure (FDR) whenever applicable (*q*-values).

### Summary statistics

We calculated median, standard deviation, and minimum and maximum values over all data points for Age, BMI, BSS, moisture content, and cycle length. Summary statistics, namely the minimum, median, and maximum values, for dietary data, such as the energy derived from fat, proteins, and carbohydrates, as well as the amount of fibers, were calculated based on the respective values for the time series data of each participant. In order to obtain the total energy intake (EI), the amount of fat, proteins and carbohydrates (g), was multiplied by 9, 4, and 4 kcal/g, respectively. Two outliers are present, one regarding a high fiber consumption (30 g/day, participant 805) and one regarding a deviating carbohydrate/fat/protein EI (with 28%, 44%, and 26% respectively, a high-fat, high-protein diet, participant 807).

### Dietary lag

In order to link the dietary and microbiome profiles, we calculated the most likely dietary lag in days for each individual. We started from a standard dietary lag of one day, the average transit time in healthy individuals^74^. Based on the relation between passage rate and moisture content, we checked which nutrient variable or combination thereof (Carbohydrates (%) (C), Proteins (%) (P), Fat (%) (F), P/C, P/F, C/F) correlated best with moisture content (mc) over all samples. We found that of all measures, the P/C ratio correlated best with mc (Pearson test, *r* = 0.23, *p*-value <10^−8^). Next, we adjusted our initial estimated lag time for each individual based on cross-correlations between this dietary measure, P/C, and mc for each individual’s time series. Therefore we determined at which lag (−1 to −5 days) the correlation between P/C and mc was highest (function *ccf*, R package *tseries*) for each individual. In case a significant correlation between P/C and mc was found with lags between 2 and 5 days, which was stronger than that observed for a 1 day lag, we adjusted the lag period accordingly (2/20 persons). In case a shorter lag period (same day) was indicated through this method, we checked if the majority of the samples were taken in the late afternoon or evening, and if so, adjusted the lag accordingly (1/20). An overview of the cross correlation analyses, relevant metadata and assigned lag period, can be found in Supplementary Table S1. All analyses regarding dietary data were performed with the lagged values.

### Fecal microbiome derived features

#### Alpha diversity

Alpha diversity measures were calculated with function *estimate_richness* of the R package *phyloseq*^75^. Pielou’s evenness index J was defined as the Shannon diversity index/log(observed richness). As the number of genera detected depends on the sequencing depth, we verified at which amount of observations richness was constant. Observed richness could be considered stable from 3 × 10^10^ bacteria/g onwards in the QMPs of the study samples, which is well below the actual number of bacteria/g within samples, which ranged from 6 × 10^10^ to 22 × 10^10^ (Supplementary Fig. 6).

#### Beta diversity

Dissimilarity between samples was calculated as Bray Curtis dissimilarity (function *vegdist*, R package *vegan*)^76^. Intra-individual dissimilarities were calculated between all samples within a time-series. Inter-individual dissimilarities were calculated using one sample of each individual, namely the one closest to the first day of menses. Within- and between-subject variability in beta-diversity were compared for each individual separately through an ANOVA test on the group dispersions through the R functions *anova* and *betadisper* (R package *vegan*). Dissimilarity over time was calculated for each pair of samples in an individual’s QMP time series belonging to the specified time category (1:36 days).

#### Enterotyping

We here define enterotypes as the states samples get assigned to when binning samples of different individuals into classes that share some similarity in microbiota composition. To bin samples into classes based on their microbiota composition several methods have been used in the past. Here, we used the Dirichlet Multinomial Mixtures (DMM) approach, as described by Holmes et al.^26^ and applied in the R package *DirichletMultinomial*^77^. This method was envisioned to be used on relative data matrices. Enterotyping was performed iteratively on a genus-abundance matrix (RMP) of 20 study samples complemented with 1103 RMPs originating from the FGFP^8^. Sample collection and sequencing protocols of both studies were similar. An iterative approach was adopted to avoid clustering two samples of the same individual at once. Each iteration included a single time point of a participant in random order.

### Mislabeling

To identify potential cases of sample mislabeling that may have occurred during preprocessing we checked whether the pairwise BC dissimilarities between a sample and all other samples attributed to the same person were lower than the pairwise BC dissimilarities between this sample and samples from every other participant in the study, with a one-sided Wilcoxon test. In our original data, 15 samples showed more similarity with samples from one or several other participants (Supplementary Data S6). In addition, we clustered the complete dataset into 20 clusters—as much as there are participants—with the DMM algorithm^26,78^, to infer the specificity of the microbiota composition of each sample. We visualized this information together with the enterotyping assignment and sample sequence (Supplementary Fig. 7). Based on all information, we identified two swapping events, one between participants 801 and 815, and another one between participant 805 and 806. We corrected these mistakes. The 11 remaining samples did not show additional indications for mislabeling and no subsequent action was undertaken. All analyses were performed on the corrected data.

### Intraclass correlation coefficient

The intraclass correlation coefficient (ICC) estimation uses the variance components from a one-way ANOVA (among-group variance and within-group variance; ICC = var_among_/[var_among_ + var_within_]). Here, the ICC was calculated based on the longitudinal QMPs, defining each individual as a distinct class, using a one-way ANOVA fixed effects model, as applied in the function *ICCest* of the *ICC* R package. Inter- and intra-personal variation was estimated using all individuals and, in the case of genus abundance, for all genera with a relative abundance>0.5% in one or more QMPs.

### Core, persistent, transient, and person-specific genera

Temporal core genera were defined as those present in more than 95% of the samples of an individual. Persistent genera were defined as those present in more than 20% of the samples with at least 90% of these observations being consecutive. Transient genera were defined as present in more than 60% of the samples while less than 75% of these observations were consecutive. Cross-sectional core genera were defined as those present in at least one sample of every individual. Person-specific genera were defined as those present in at least one sample of a person while absent in all other individuals, excluding those detected within the negative controls. Analyses were carried out based on QMPs for all genera with a relative abundance>0.5% in the QMPs.

### Fold changes in genus abundance

Here, an x-fold change should be interpreted as x times. Maximum fold changes over the complete study period or between consecutive samples were calculated per individual, for every detected genus above the abundance threshold, on QMPs.

### Stationarity

To test whether genera varied around an equilibrium level in a participant or showed non-stationary behavior, we used the Augmented Dickey–Fuller test (ADF)^79^. A significant ADF test suggests non-stationary behavior as it rejects the existence of a unit root process. We applied the ADF test on the QMPs of an individual through function *adf.test* of the R package *aTSA*^80^. We limited the investigated genera to those present in more than 60% of the samples.

### Mean–variance relationships (Taylor’s law, TL)

TL states that *log(variance)*_*g*_ = *b × log(mean)*_*g*_, where *b* is a species-specific constant and *g* indicates the group of measurements. For a given unit change in the log(mean) of population abundance, the TL’s slope equals the change in log(variance), which measures heterogeneity or scatter in the distribution of population abundance. A greater slope in the temporal TL means a greater degree of change in the temporal variance of genus population abundance with respect to its temporal mean. Genera for which the TL slope is 2 have about the same coefficient of variation in all subjects. Genera for which the TL slope is less than 2 show more temporal variation in subjects in which they are more abundant. The reverse for genera for which the TL slope is greater than 2. For each genus, the linearity of the relationship between log(variance) and log(mean) over time was tested using function *lm*, calculating slopes, *p*-values and adjusted *R*^2^ (Supplementary Table S2). In accordance with literature^28^, we removed time series with fewer than three measurements of the genus, to ensure that the variance was properly defined. We excluded genera which were present in fewer than 5 individuals, to avoid slope determination based on only a few data points.

### Error on median genus abundances with increasing timepoints

Standard error on median genus abundance for a given sample size was calculated by randomly subsampling the time series at different levels of temporal resolution (1–21 timepoints), for the 100 most-abundant genera of each participant, over all participants, with 10.000 repetitions.

### Principal coordinates analysis (PCoA)

Microbiome inter and intra-individual variation and individual trajectories were visualized by PCoA using Bray–Curtis dissimilarity on a combined matrix including all genus-level RMPs of this study (709 samples) together with those of the FGFP (1103 samples) (R package *vegan*).

### Similarity decay analysis

According to similarity decay analysis^81^, the slope of a log-linear model fitted between the change in community structure and the time elapsed represents the rate of community change over time. We performed the analysis for of all non-perturbed time series (*N* = 19, individual 808 excluded) and limited it to 36 days, to include data of each individual at every day, randomly picking time points so that each day contained an equal number of data points (*n* = 3780, 105/time point). Community dissimilarities (BC) between samples of QMP time series were converted to similarities by subtracting from one. Log-transformed similarities were set out against the number of days elapsed. To visualize the model, we applied *lowess* smoothing over windows the length of 5% of the total series.

### Estimation of the community variation explained by metadata variables

Variation partitioning by distance-based redundancy analysis (dbRDA) was performed to determine how much of the microbial community profiles variation could be explained independently of other covariates by (i) the individual, with model M-null= BC ≈ ID-number and (ii) the metadata, with null model M-null and alternative model M-alt= BC ≈ ID-number + metadata variable. A stepwise dbRDA was performed to estimate the cumulative effect of the metadata variables correcting for individual, with null model M-null= BC ≈ ID-number and alternative model M-alt-step= BC ≈ ID-number + all metadata variables; with BC the Bray Curtis dissimilarity matrix on QMPs, using functions *vegdist*, *capscale* and *ordiR2step* of R package *vegan*. We performed the analysis for of all non-perturbed time series (*N* = 19, individual 808 excluded).

### Enterotype shifts and compositional dissimilarity

We used a Chi square test goodness of fit with 10 categories, one for each enterotype switch (B2-B2, R-R, B1-B1, B2-B1, R-B1, P-P, P-R, B2-R, P-B1, B2-P), in order to test whether the observed number of enterotype switches was in line with what could be expected based on enterotype prevalence. For each transition, within or between enterotypes, we calculated the expected probability of an A-to-B transition as: the number of A-to-B transitions possible within the study dataset / total number possible transitions within the study dataset. In this way we took into account that samples were differently distributed over the enterotypes. Standardized residuals of the Chi square test were used as a measure for the discrepancy between observed numbers and expected transition rates.

The average compositional dissimilarity between states was calculated as the median BC dissimilarity between samples of the respective states, considering all samples of the study cohort. Significant differences in compositional dissimilarity between states were assessed using a Dunn test. Given the longitudinal nature of the study data, the thus for described compositional dissimilarities include within- and between-subject variability. In order to disentangle both components we calculated the between- and within-subject compositional dissimilarity separately for each enterotype. Between-subject compositional variabilities of an enterotype were calculated using the cross-sectional data of the FGFP cohort (*N* = 1103), as the BC dissimilarity between samples of different individuals within the same enterotype. Differences in dispersion across enterotypes were assessed through an ANOVA test on the group dispersions through the R functions *anova* and *betadisper* (R package *vegan*) Within-subject compositional variabilities were calculated using the longitudinal study cohort data, excluding participant 808, due to an unstable microbial community after an infection event (*N* = 19), as the median BC dissimilarity between samples of the same individual. These within-subject compositional dissimilarity measures were grouped according to enterotype, based on the enterotype label of the majority of an individual’s samples. Significant differences in within-subject compositional dissimilarity between enterotypes were assessed using a Dunn test (function *dunn.tes*t from R package *dunn.test*).

### Enterotype shifts and associations with metadata

To find possible metadata triggers for enterotype switches we applied multi-state transition modeling (non-hidden Markov Model) with covariates using the R package *msm*^41^. We defined the transition model using the function *msm*, with enterotypes as states, days as time-information and ID-number as subject information, with the option qmatrix set to the crude transition matrix based on the observed data and equal transition probabilities for all shifts, and the option deathexact set to FALSE. The significance of the independent contribution of the metadata variable to the transition model was tested using a log likelihood ratio test (function *lrtest.msm*) comparing the model with and without the metadata variable. Risk ratios and 95% confidence intervals were calculated for each metadata variable (function *hazard.msm*) and visualized in a forest plot (R package *ggplot2*). The effect of a metadata variable on a specific enterotype transition was considered significant (*P* < 0.05) if the 95% CI of the hazard ratio did not include 1.

### Characterization of within and between-subject effects of metadata on community characteristics or genus abundance

#### Over all individuals

Linear models were used to assess metadata-community characteristics or metadata-genus abundance correlations over all individuals. Analyses on genus abundance were limited to those genera satisfying the abundance and prevalence thresholds (genus present in >5 participants and >10 samples per participant, with median relative abundance over all QMPs in which it was detected>0.5%). A General Linear Model (GLM) is a flexible generalization of ordinary linear regression model (LM) for response variables with error distribution models other than the normal distribution, which was necessary as genus abundance data were best fitted by a negative binomial distribution. We used GLMs to assess the between-subject correlation between variables and taxon abundance, using the first data point of each time series (model 1). A GLMM is a GLM with the possibility to vary the intercept and slope of the regression according to a specified random effect. A model treating ID as a random effect for the intercept allows the intercept to take a different value per person according to its mean genus abundance and thus takes into account the temporal variation between subjects (model 2). A model treating ID as a random effect for both the intercept and slope in addition allows correlation coefficients to vary according to individual and thus takes into account not only the temporal variation between subjects but also within subjects (model 3). We included all collected data points within the GLMMs (models 2 and 3). We defined the different GL(M)Ms, as follows: [model 1] glm1 = Gi ≈ variable, [model 2] glmm2 = Gi ≈ variable + (1|ID), and [model 3] glmm2 = Gi ≈ variable + (1+variable|ID), where ID is the person identifier, and Gi is the quantitative genus abundance matrix, with ‘i’ being the genus index, using function *glm.nb* and *glmer.nb* of the *lme4* R package with default values^82^. We checked correlation (Pearson test) between all metadata variables and excluded redundant variables for pairs where *r* > 0.2. We standardized metadata variables and expressed genus abundances in percentage prior to modeling in order to be able to compare relative contribution of each variable by the estimates. To assess the temporal effect of the metadata on microbiota measurements, such as microbial load and alpha diversity measurements, a similar approach was used. We defined the L(M)Ms as follows: [model 1] lm1 = MM ≈ variable, [model 2] lmm2 = MM ≈ variable + (1|ID), and [model 3] lmm3 = MM ≈ variable + (1+variable|ID), where MM is the microbiota measurement of interest, using functions *lm* and *lmer* of the *lme4* R package with default values^82^. In order to assess the effect of metadata variables on taxon abundance beyond the effect of moisture content, we defined the GLMM as follows: [model 4] glmm4 = Gi ≈ variable + (1+variable|ID) + (1+variable|MC), where ID is the person identifier, MC is the moisture content, and Gi is the quantitative genus abundance matrix, with ‘i’ being the genus index.

#### Per individual

Correlations between continuous variables were analyzed using non-parametric Spearman tests.

### Reporting summary

Further information on research design is available in the Nature Research Reporting Summary linked to this article.

## Supplementary information

Supplementary Information

Description of Additional Supplementary Files

Supplementary Dataset 1

Supplementary Dataset 2

Supplementary Dataset 3

Supplementary Dataset 4

Supplementary Dataset 5

Supplementary Dataset 6

Reporting Summary

## Data Availability

Supplementary Information is available in the online version of the paper. Supplementary Data S1 contains the QMP, RMP, and non-rarefied profiles of the samples and controls, the taxonomic table, and the microbiome derived data. Raw amplicon sequencing data that support the findings of this study have been deposited in the European Genome-Phenome Archive with accession code EGAS00001005686 with public access. Source data for all figures are provided with the paper. Additional data requests can be directed to the corresponding author. Source data are provided with this paper.

## Source data

Source Data

## Code availability

An updated open source QMP R-script is available on http://www.raeslab.org/software/QMP temporal and the Github repository (https://github.com/raeslab/temporal_variability_microbiome_qmp) with public access.
