# Supplementary material for: Temporal variability in quantitative human gut microbiome profiles and implications for clinical research
Source: Nat Commun. 2021 Nov 18;12:6740. doi: 10.1038/s41467-021-27098-7 (PMC8602282; doi:10.1038/s41467-021-27098-7)
Supplement: Supplementary file 1 — Supplementary Information [file 41467_2021_27098_MOESM1_ESM.pdf]

SUPPLEMENTARY INFORMATION

Temporal variability in quantitative human gut microbiome profiles and implications for clinical research.

Doris Vandeputte, Lindsey De Commer, Raul Y. Tito, Gunter Kathagen, João Sabino, Séverine Vermeire, Karoline Faust, and Jeroen Raes.§

Content

Supplementary Figure 1
Supplementary Figure 2
Supplementary Figure 3
Supplementary Figure 4
Supplementary Figure 5
Supplementary Figure 6
Supplementary Figure 7
Supplementary Figure 8

Supplementary Table S1
Supplementary Table S2

SUPPLEMENTARY FIGURES AND TABLES

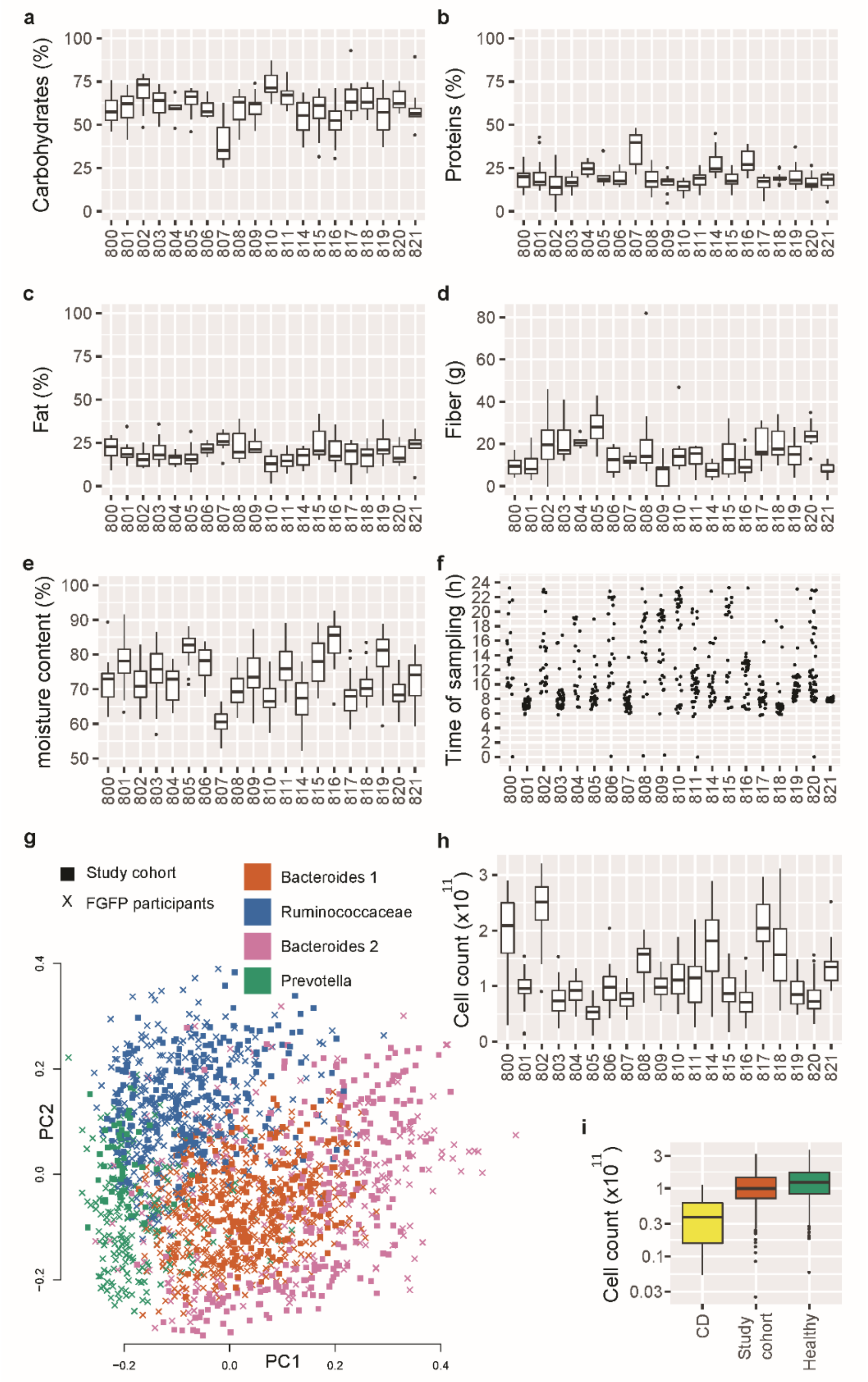

Supplementary Figure 1: **Overview of study cohort characteristics and variation in metadata.** Box plots of percentage of total weight of food products consumed per day for **a.** Carbohydrates (ICC: 0.21), **b.** Proteins (ICC: 0.40), and **c.** Fat (ICC: 0.21), for each participant. **d.** Total weight of fiber consumed per day (ICC: 0.32), for each participant. **e.** Box plots of stool moisture content (%) for each participant (ICC:0.51). **f.** Time of stool sampling according to a 24h-scale, for each participant. **g.** Principal Coordinate Analyses of RMPs of all study cohort samples (squares) and 1104 FGFP participants (crosses),

coloured according to enterotype, namely Ruminoccocaceae (R, blue), Bacteroides 1 (B1, orange), Bacteroides 2 (B2, Pink), Prevotella (P, green). **h.** Box plots of microbial load, in cell counts per gram of faeces, for each participant (ICC:0.61). **i.** Microbial load, in cell counts per gram of faeces, of all samples (Study Cohort) in comparison with previously sampled cohorts of Crohn's Disease patients (CD) and healthy volunteers (Healthy)[11].
The body of the box plots within these figures represents the first and third quartiles of the distribution, and the median line. The whiskers extend from the quartiles to the last data point within 1.5× IQR, with outliers beyond. Figures and summary measures are based on all data (N=20, n=713) (Supplementary Table S1 & S2).

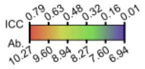

	QMP					Coeff. Variation Within			Coeff. Variation	RMP Coeff. Variation Within			RMP Coeff. Variation
	Slope TL	Core	Pers.	Trans.	Non-St.	Q1	Q2	Q3	Between	Q1	Q2	Q3	Between
g_Faecalibacterium (Firm)	1.6	20	20	0	5	0.6	0.7	0.8	0.5	0.3	0.3	0.5	0.5
g_Roseburia (Firm)	1.7	20	19	0	3	0.7	0.8	0.9	0.7	0.4	0.5	0.7	0.7
g_Bacteroides (Bact)	1.9	20	20	0	2	0.6	0.7	0.8	0.5	0.3	0.4	0.5	0.5
g_Alistipes (Bact)	1.7	20	20	0	1	0.6	0.7	1	0.9	0.4	0.5	0.8	0.9
uc_f_Lachnospiraceae (Firm)	1.7	20	20	0	3	0.5	0.6	0.7	0.4	0.3	0.3	0.4	0.4
g_Clostridium_IV (Firm)	1.8	17	16	4	4	0.7	0.7	0.8	1	0.5	0.6	0.7	1
g_Blautia (Firm)	2	20	20	0	2	0.6	0.7	0.8	0.8	0.4	0.4	0.5	0.8
g_Anaerostipes (Firm)	1.9	19	19	0	5	0.6	0.7	0.8	0.8	0.4	0.5	0.8	0.8
uc_f_Ruminococcaceae (Firm)	1.7	20	19	1	2	0.6	0.7	0.7	0.9	0.3	0.4	0.5	0.9
g_Ruminococcus2 (Firm)	1.7	20	20	0	3	0.6	0.7	0.8	0.9	0.4	0.5	0.6	0.9
g_Prevotella (Bact)	1.7	6	4	2	0	1.1	1.7	3	2.2	1.1	2.3	5.3	2.2
g_Ruminococcus (Firm)	1.8	17	17	1	2	0.7	0.8	0.9	0.7	0.5	0.5	0.6	0.7
g_Bifidobacterium (Acti)	1.7	12	12	3	2	0.9	1	1.2	1.5	0.7	0.8	1.1	1.5
g_Sutterella (Prot)	1.9	15	15	0	0	0.8	0.8	1	0.8	0.5	0.6	0.8	0.8
g_Clostridium_XlVa (Firm)	1.9	20	18	1	3	0.6	0.7	0.9	0.6	0.4	0.5	0.6	0.6
uc_o_Clostridiales (Firm)	1.7	14	14	3	3	0.7	0.8	0.9	1	0.4	0.5	0.7	1
g_Coprococcus (Firm)	1.8	15	15	2	3	0.6	0.8	0.9	1	0.4	0.5	0.7	1
g_Fusicatenibacter (Firm)	1.7	17	17	1	3	0.7	0.8	0.9	0.7	0.5	0.5	0.6	0.7
g_Oscillibacter (Firm)	1.7	17	15	4	1	0.7	0.8	0.9	0.7	0.4	0.6	0.7	0.7
uc_f_Rhodospirillaceae (Prot)	1.7	5	4	2	0	1	1.2	1.5	2.8	0.9	1	1.4	2.8
g_Dorea (Firm)	1.6	18	17	2	4	0.6	0.6	0.8	0.8	0.3	0.4	0.5	0.8
uc_k_Bacteria (Unkn)	1.6	9	8	3	0	1	1.3	2.8	1.4	0.7	1	1.8	1.4
uc_p_Firmicutes (Firm)	1.6	8	7	5	2	0.8	1	1.3	1.2	0.6	0.8	1.1	1.2
g_Collinsella (Acti)	2	12	9	8	1	0.9	1	1.1	1	0.7	0.9	1	1
g_Gemmiger (Firm)	1.8	15	13	5	2	0.7	0.9	1.1	0.8	0.5	0.6	0.7	0.8
uc_f_Porphyromonadaceae (Bact)	1.5	5	4	5	3	0.8	0.9	1.4	3.3	0.5	0.7	1	3.3
g_Akkermansia (Verr)	1.8	8	7	6	0	1.1	1.6	2	1.3	1	1.3	1.9	1.3
g_Clostridium_XVIII (Firm)	1.8	13	10	7	2	0.8	0.9	1	0.9	0.6	0.7	0.9	0.9
g_Parabacteroides (Bact)	2.4	15	12	5	2	0.7	0.8	0.9	0.8	0.5	0.6	0.8	0.8
g_Dialister (Firm)	1.7	9	8	1	1	0.7	0.8	0.9	1.6	0.5	0.5	0.7	1.6
g_Desulfovibrio (Prot)	1.4	5	5	2	0	0.6	0.8	0.8	3.2	0.4	0.5	0.5	3.2
g_Sporobacter (Firm)	1.6	11	8	6	2	0.7	0.8	1	0.9	0.5	0.6	0.8	0.9
g_Butyricicoccus (Firm)	1.6	14	10	6	1	0.7	0.8	0.9	0.5	0.5	0.6	0.7	0.5
g_Elusimicrobium (Elus)	·	2	2	0	1	·	·	·	·	·	·	·	·
g_Barnesiella (Bact)	1.8	13	11	3	2	0.8	0.8	1	1	0.6	0.6	0.7	1
uc_f_Coriobacteriaceae (Acti)	1.7	5	3	8	0	1	1.2	1.4	2.1	0.9	1	1.1	2.1
g_Parasutterella (Prot)	1.4	5	5	5	1	0.8	1	1.3	1.5	0.6	0.8	1.1	1.5
g_Aestuariispira (Prot)	1.7	4	3	2	1	1	1.5	2.1	2.4	0.9	1.2	1.6	2.4
g_Paraprevotella (Bact)	2.4	7	6	2	0	0.8	0.9	1	2.1	0.6	0.6	0.8	2.1
g_Adlercreutzia (Acti)	1.3	7	7	5	1	0.7	0.9	1.3	1.1	0.8	0.9	1.4	1.1
g_Hespellia (Firm)	1.6	4	3	7	2	0.8	1	2	2.4	0.5	0.7	1.6	2.4
g_Streptococcus (Firm)	1.6	1	1	10	0	1.1	1.5	1.9	2.7	1	1.2	1.4	2.7
g_Butyrivibrio (Firm)	·	1	1	0	0	·	·	·	·	·	·	·	·
g_Bilophila (Prot)	1.6	7	6	6	1	0.8	0.9	1.1	0.8	0.5	0.6	0.8	0.8
g_Clostridium_XlVb (Firm)	1.4	5	3	10	0	0.8	1	1.2	1	0.6	0.7	0.8	1
g_Butyricimonas (Bact)	1.8	4	2	12	0	0.9	0.9	1.1	0.9	0.5	0.6	0.7	0.9
uc_f_Rikenellaceae (Bact)	1.4	3	1	2	0	0.8	1.9	3.2	3.3	0.5	1.8	2.7	3.3
g_Intestinimonas (Firm)	1.5	6	4	11	4	0.8	0.9	1.1	0.7	0.5	0.6	0.8	0.7
g_Flavonifractor (Firm)	1.4	5	1	7	1	0.9	1.2	1.9	1.2	0.8	0.9	1.8	1.2
g_Senegalimassilia (Acti)	1.5	3	1	2	0	0.8	1.1	1.3	1.9	0.8	0.9	1.1	1.9
g_Odoribacter (Bact)	1.5	4	2	12	0	0.9	1	1.1	0.7	0.6	0.6	0.8	0.7
uc_c_Alphaproteobacteria (Prot)	·	1	1	1	1	·	·	·	·	·	·	·	·
g_Phascolarctobacterium (Firm)	1.9	5	4	4	3	0.7	0.8	1.2	1.5	0.5	0.6	0.7	1.5
g_Slackia (Acti)	1.4	2	1	5	0	1	1.1	1.6	1.9	0.9	1.2	1.7	1.9
g_Methanobrevibacter (Eury)	2.2	1	0	8	0	1	1.1	1.4	1.3	0.8	1.1	1.5	1.3
uc_f_Sutterellaceae (Prot)	·	2	2	0	0	·	·	·	·	·	·	·	·
g_Cerasicoccus (Verr)	1.5	2	2	2	1	1.1	1.6	2.6	2.4	1	1.2	2.4	2.4
g_Hydrogenoanaerobacterium (Firm)	1.3	3	2	7	1	0.9	1	1.2	1.5	0.8	0.9	1.3	1.5
g_Coprobacter (Bact)	1.4	2	1	7	0	1	1.2	1.5	1.3	0.7	0.9	1.1	1.3
g_Victivallis (Lent)	1.6	0	0	9	0	1.1	1.2	1.4	1.9	0.9	0.9	1.2	1.9
uc_f_Erysipelotrichaceae (Firm)	1.4	1	3	2	0	1.1	1.4	2.5	2.6	1.1	1.3	2.7	2.6
g_Subdoligranulum (Firm)	1.5	0	1	1	1	1.5	2.5	3.9	2.3	1.4	2	3.8	2.3
g_Veillonella (Firm)	1.4	1	1	0	0	1.7	4	4.9	3.6	1.7	3.1	4.9	3.6
uc_p_Proteobacteria (Prot)	1.6	2	0	3	0	1.2	1.4	1.7	2.1	0.9	1.4	2.2	2.1
g_Holdemanella (Firm)	·	2	2	1	0	·	·	·	·	·	·	·	·
uc_c_Clostridia (Firm)	1.2	2	2	4	1	0.8	1.4	3.2	1.6	0.7	0.9	2.3	1.6
g_Vampirovibrio (Prot)	·	1	1	0	0	·	·	·	·	·	·	·	·
g_Olsenella (Acti)	1.7	1	1	2	0	1	1.1	1.3	2.2	0.9	1	1.2	2.2
g_Pseudoflavonifractor (Firm)	1.3	0	0	2	0	1.6	2	2.6	1.8	1.2	1.6	2.4	1.8
g_Anaerosporobacter (Firm)	1.3	2	2	3	0	1.1	2.1	4.7	1.5	0.9	1.8	3.2	1.5
g_Intestinibacter (Firm)	1.2	0	0	3	0	1.2	1.5	2.3	1.2	0.9	1.1	1.8	1.2
uc_o_Rhodospirillales (Prot)	·	1	0	1	0	·	·	·	·	·	·	·	·
g_Haemophilus (Prot)	1.4	0	0	3	0	1.5	2.3	3.5	2.1	1.3	2.1	3	2.1
g_Faecalicoccus (Firm)	1.3	0	0	5	2	1.2	1.6	2.8	1.7	0.9	1.7	2.2	1.7
g_Alloprevotella (Bact)	·	1	1	0	1	·	·	·	·	·	·	·	·
g_Allobaculum (Firm)	·	0	0	0	0	·	·	·	·	·	·	·	·
g_Rikenella (Bact)	·	1	1	0	0	·	·	·	·	·	·	·	·
g_Romboutsia (Firm)	1.3	0	0	5	0	1.3	1.5	2.1	0.9	1.2	1.4	2	0.9
g_Catenibacterium (Firm)	·	2	1	1	0	·	·	·	·	·	·	·	·
g_Desulfomicrobium (Prot)	·	1	1	0	0	·	·	·	·	·	·	·	·
g_Enterorhabdus (Acti)	1.5	0	0	4	0	1	1.2	1.4	2.6	0.8	0.9	1.2	2.6
g_Faecalitalea (Firm)	·	1	1	0	0	·	·	·	·	·	·	·	·
g_Escherichia.Shigella (Prot)	1.9	0	1	0	0	2.2	2.8	3.6	1.7	1.6	2.5	3.1	1.7
uc_c_Deltaproteobacteria (Prot)	·	0	0	1	0	·	·	·	·	·	·	·	·
uc_p_Bacteroidetes (Bact)	1.3	1	0	1	0	1.1	1.5	2	2.1	1	1.4	5	2.1
g_Eggerthia (Firm)	·	0	0	0	0	·	·	·	·	·	·	·	·
g_Anaerotruncus (Firm)	1.2	1	1	1	1	1.5	3	3.5	1.8	1.6	2.7	3.2	1.8
g_Oligosphaera (Lent)	·	0	0	2	0	·	·	·	·	·	·	·	·
uc_f_Desulfovibrionaceae (Prot)	·	0	1	0	0	·	·	·	·	·	·	·	·
uc_o_Burkholderiales (Prot)	·	1	0	1	0	·	·	·	·	·	·	·	·
g_Gordonibacter (Acti)	1.4	0	0	1	0	2.7	3.5	4.5	3	2.9	3.5	5.4	3
uc_f_Prevotellaceae (Bact)	·	1	1	0	0	·	·	·	·	·	·	·	·
uc_f_Enterobacteriaceae (Prot)	·	0	0	0	0	·	·	·	·	·	·	·	·
g_Methanomassiliicoccus (Eury)	·	0	0	2	0	·	·	·	·	·	·	·	·
g_Enterococcus (Firm)	·	0	0	0	0	·	·	·	·	·	·	·	·
g_Lactobacillus (Firm)	·	0	0	0	0	·	·	·	·	·	·	·	·

Supplementary Figure 2. Variation and core measurements for all genera.
For all genera above the abundance threshold: (Ab.) Log mean abundance of the genus over all samples in which it was present; (ICC) Intraclass correlation coefficient of the genus; (Slope TL) Slope of Taylor's law for the genus calculated over all individuals (1.6±0.25 [1.2-2.4]); (Core) The number of participants for which the genus belongs to the core, defined as present in more than 95% of the samples; (Pers.) The number of participants for which the genus can be considered persistent, defined as present in more than 20% of the samples with at least 90% of these observations being consecutive; (Trans.) The number of participants for which the genus can be considered transient, defined as present in more than 60% of the samples while less than 75% of these observations are consecutive; (Non-St.) The number of participants in which the genus shows non-stationary dynamics, defined as not satisfying a unit root process in which abundances vary around an equilibrium level (Augmented Dicky Fuller test, p>0.05). (Coef. Variation) Coefficient of variation in genus abundance within and between individuals. The coefficient of variation is defined as the ratio of the standard deviation to the mean and is a standardized measure of dispersion. Within-subject variation is summarized as the first, second and third quartile (Q1,Q2,Q3, respectively) of all values for the within-subject coefficient of variation, calculated for each time series (N=20). Data are based on either quantitative (QMP) or relative (RMP) microbiome profiling.

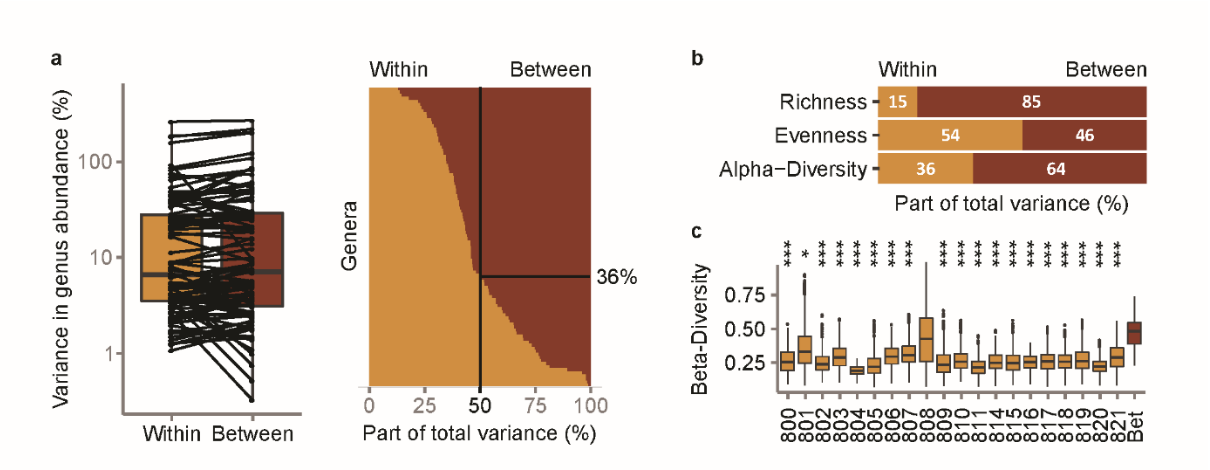

Supplementary Figure 3. Within- compared to between-subject variance for relative abundance profiles. a. Within- versus between-subject variance in relative genus abundance (Wilcoxon test, two-sided, n=98, P= 0.007154) (left) and within-subject and between-subject variance as part of the total variance in genus abundance (right) for all genera over the abundance threshold. **b.** Within- and between-subject variance as part of the total variance in alpha diversity measures, for observed richness, Pielou evenness, and Shannon diversity index based on RMPs. **c.** Beta-diversity, as assessed through Bray Curtis dissimilarity, between samples of the same individual (800-821), and between the first sample of each individual (Bet) based on RMPs (N=20, n=709),. Significance of the differences within and between individuals were tested through an ANOVA on multivariate homogeneity of group dispersions.

The body of the box plots represents the first and third quartiles of the distribution, and the median line. Whiskers extend from the quartiles to the last data point within 1.5×IQR, with outliers beyond. Significance levels: ***: 0.001, **: 0.01, *: 0.05.

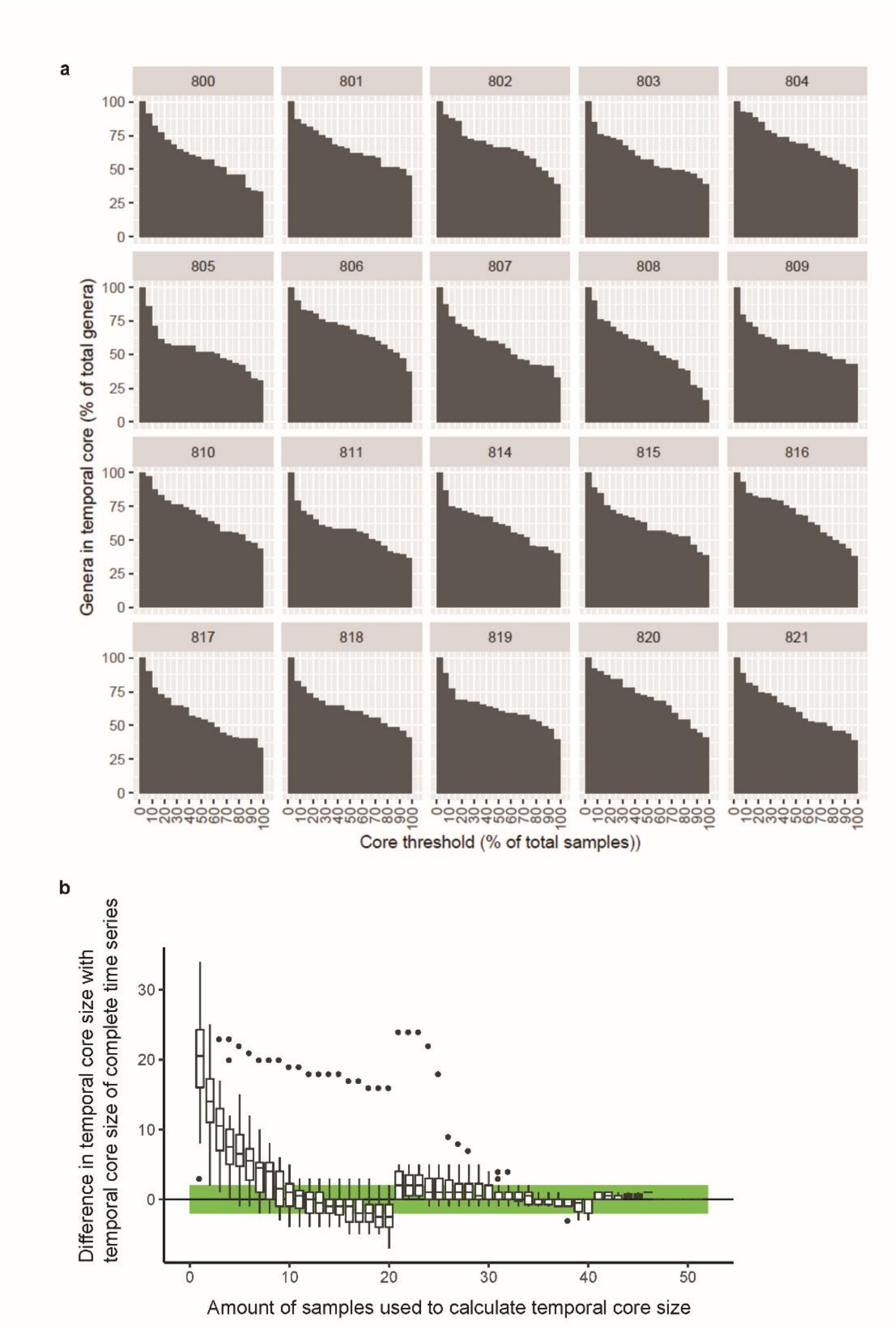

Supplementary Figure 4. a. Percentage of genera belonging to the temporal core, according to different core thresholds, for each individual. With a core threshold of 90%, about 44% [27-52%] of a person's genera are included. With less stringent temporal core thresholds more genera satisfy the conditions (e.g. about 73% [57-86%] of a person's genera are detected more than 20% of the time (the threshold for the persistent category),

and about 58% [48-69%] of a person's genera are detected more than 60% of the time (the threshold for the transient category). **b. Temporal core size falls within 90% of the all-samples estimate from 11 samples onwards for all non-perturbed time series.** For increasing lengths of time series (x-axis) we calculated the temporal core size (with a 95% threshold, which explains the stepwise increase after 20 and 40 samples, N=20, n=694) and compared it with the temporal core size obtained if all samples were to be included (y-axis). The time series of participant 808, which included an infection event, is responsible for the major outliers.

The body of the box plots represents the first and third quartiles of the distribution, and the median line. Whiskers extend from the quartiles to the last data point within 1.5×IQR, with outliers beyond. Significance levels: ***: 0.001, **: 0.01, *: 0.05.

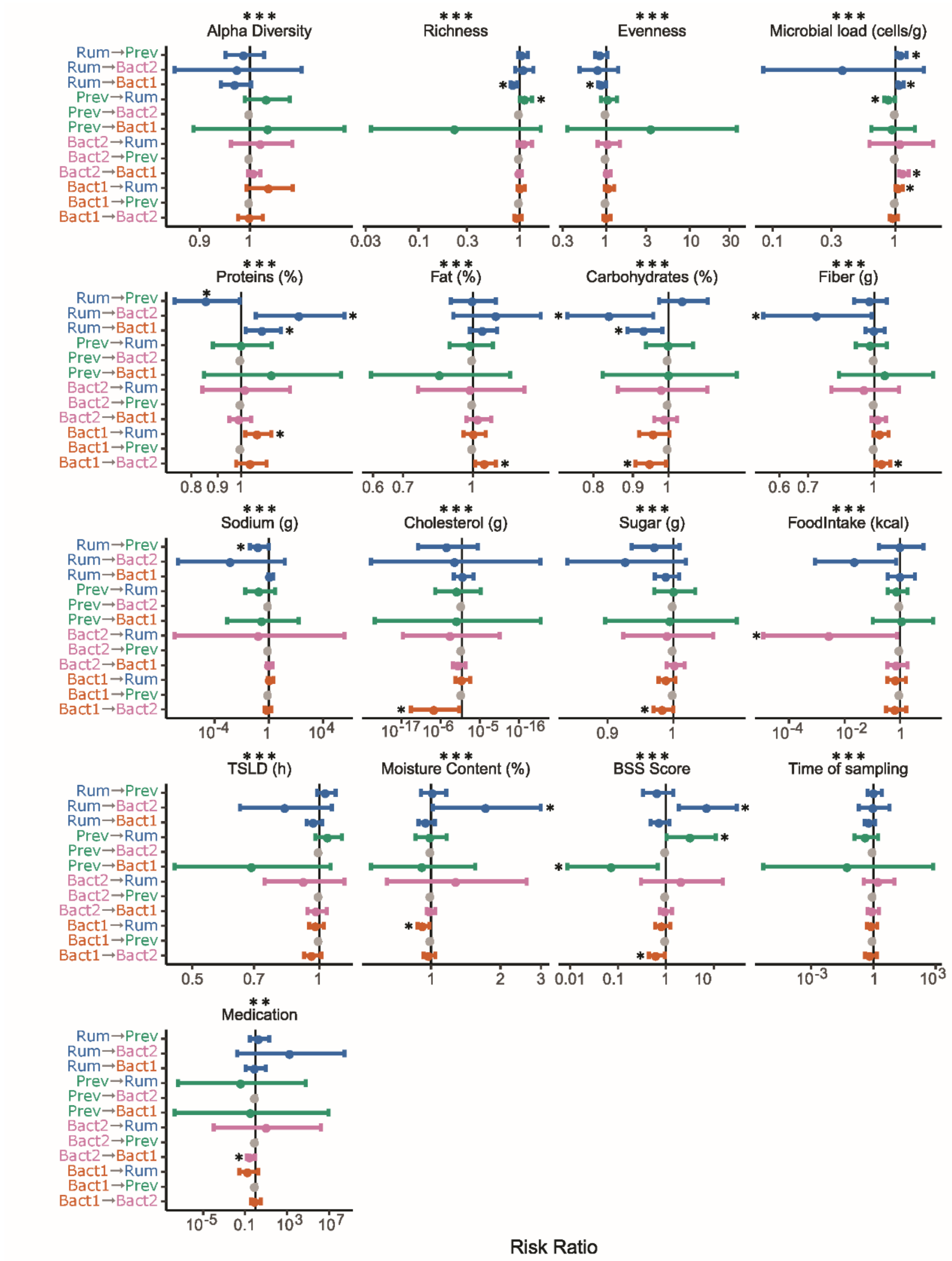

Supplementary Figure 5. Enteroype switches are linked to several investigated parameters. The relative risk of an enterotype switch with each unit increase in the parameter (risk ratio, x-axis), set out for each possible enterotype switch (y-axis). Switches are color coded according to data availability: no data (grey), and subsequently to direction of the switch: away from the Ruminoccocaceae (R, blue), Bacteroides 1 (B1, orange), Bacteroides 2 (B2, Pink), Prevotella (P, green) enterotype (N=20, n=709). Error bar dots indicate the median value with whiskers extending to the 95% confidence interval. Significance levels: ***: 0.001, **: 0.01, *: 0.05.

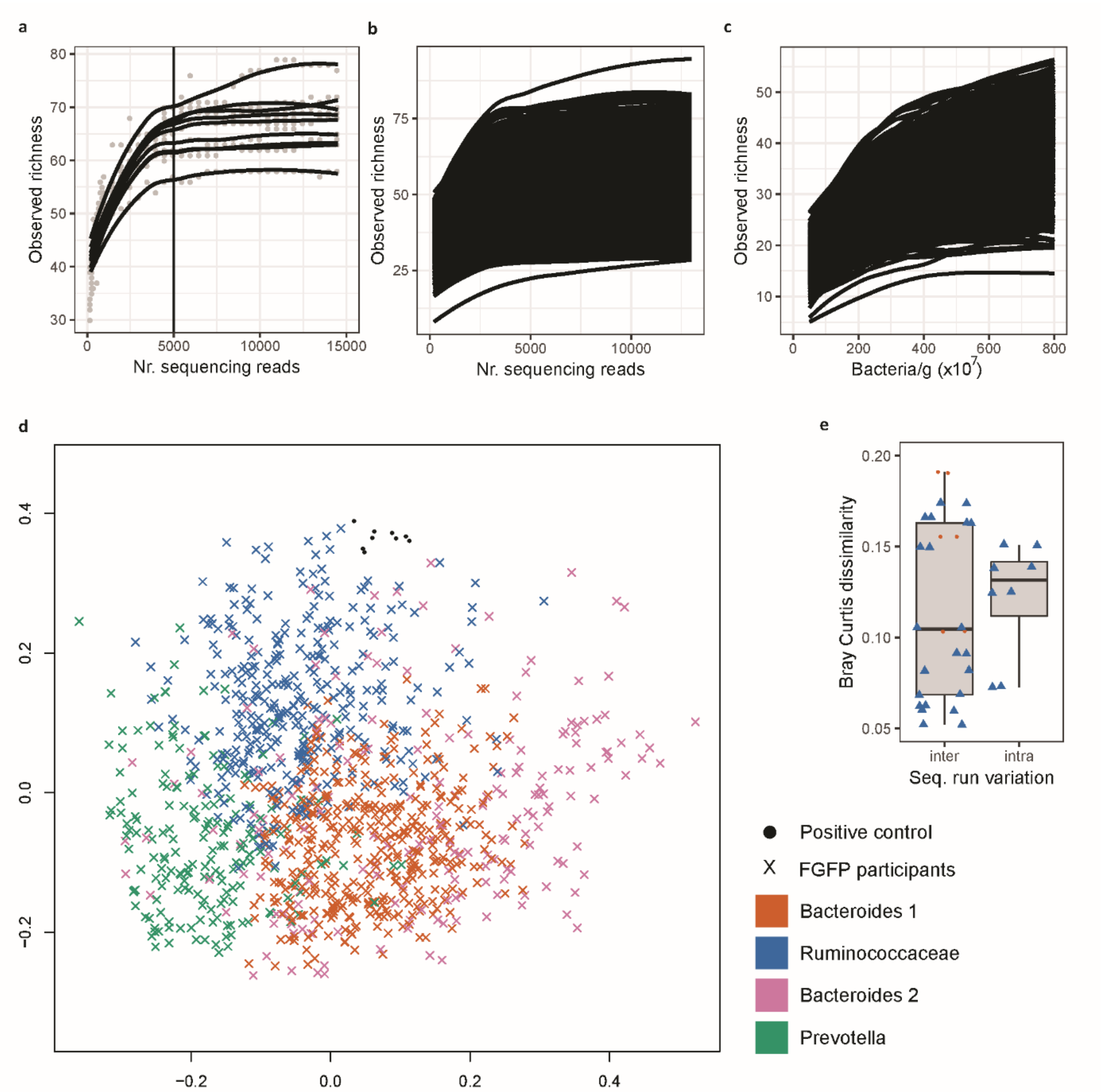

Supplementary Figure 6. Quality control of sequencing data. Richness could be considered stable in **a.** controls (n=9) and **b.** RMPs (N=20, n=709), from 5000 reads, and in **c.** QMPs (N=20, n=694), from $4x10^9$ bacterial cells per gram of faeces, which is well below the applied subsampling thresholds. **d.** PCoA of the positive control samples (black points) together with the RMPs of 1103 FGFP participants (crosses), coloured according to enterotype, namely Ruminoccocaceae (R, blue), Bacteroides 1 (B1, orange), Bacteroides 2 (B2, Pink), Prevotella (P, green). **e.** Bray Curtis Dissimilarity between positive control samples, derived from 2 aliquots of DNA from the same high-richness stool sample (orange dots, blue triangles), between (inter) and within (intra) sequencing runs (n=9).

The body of the box plots represents the first and third quartiles of the distribution, and the median line. Whiskers extend from the quartiles to the last data point within 1.5×IQR, with outliers beyond. Significance levels: ***: 0.001, **: 0.01, *: 0.05.

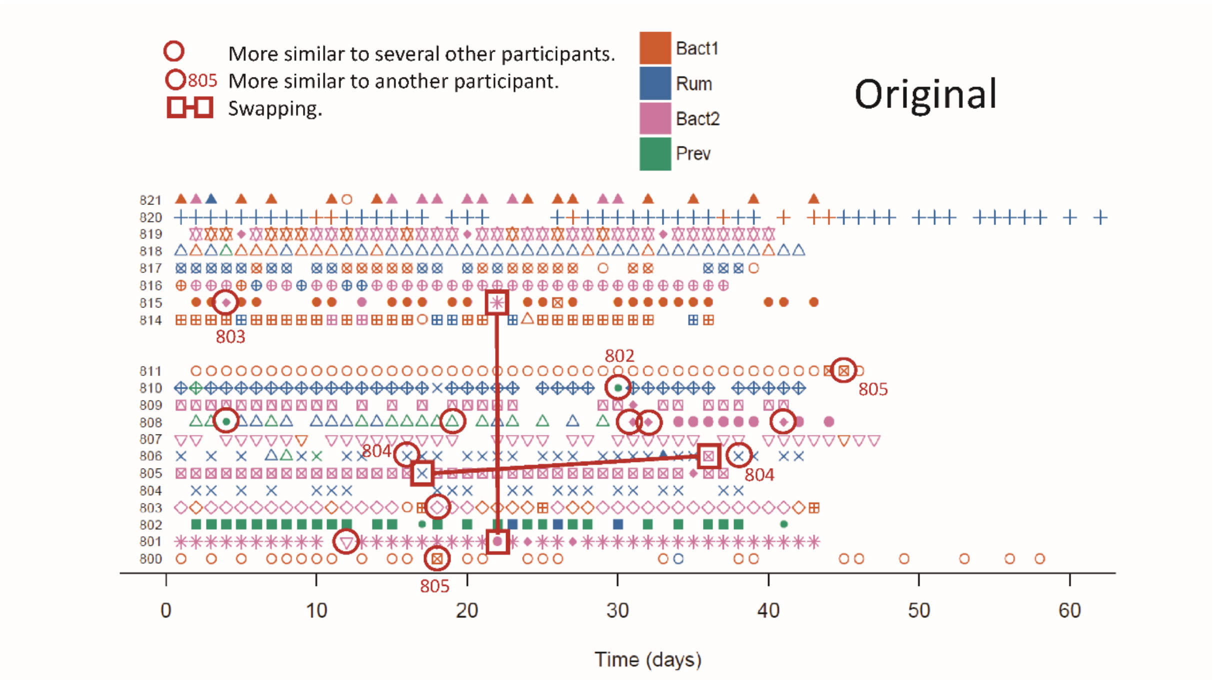

Supplementary Figure 7 Graphical presentation of the mislabelling analyses and corrections. Two swapping events were identified based on comparisons between BC dissimilarities of a sample with those of same or different participants, and DMM-clustering. For each participant (800-821), samples are shown according to sampling moment with an indication of enterotype, namely, Ruminococcaceae (blue), Bacteroides 1 (orange), Bacteroides 2 (Pink), and Prevotella (green), and the assigned DMM sub-cluster (20 shapes). In our original data, 15 samples showed more similarity with samples from one (circles with participant number) or several other participants (circle). Swapping events (connected squares) were identified based on according mismatches in BC-dissimilarities, enterotyping, and DMM-clustering.

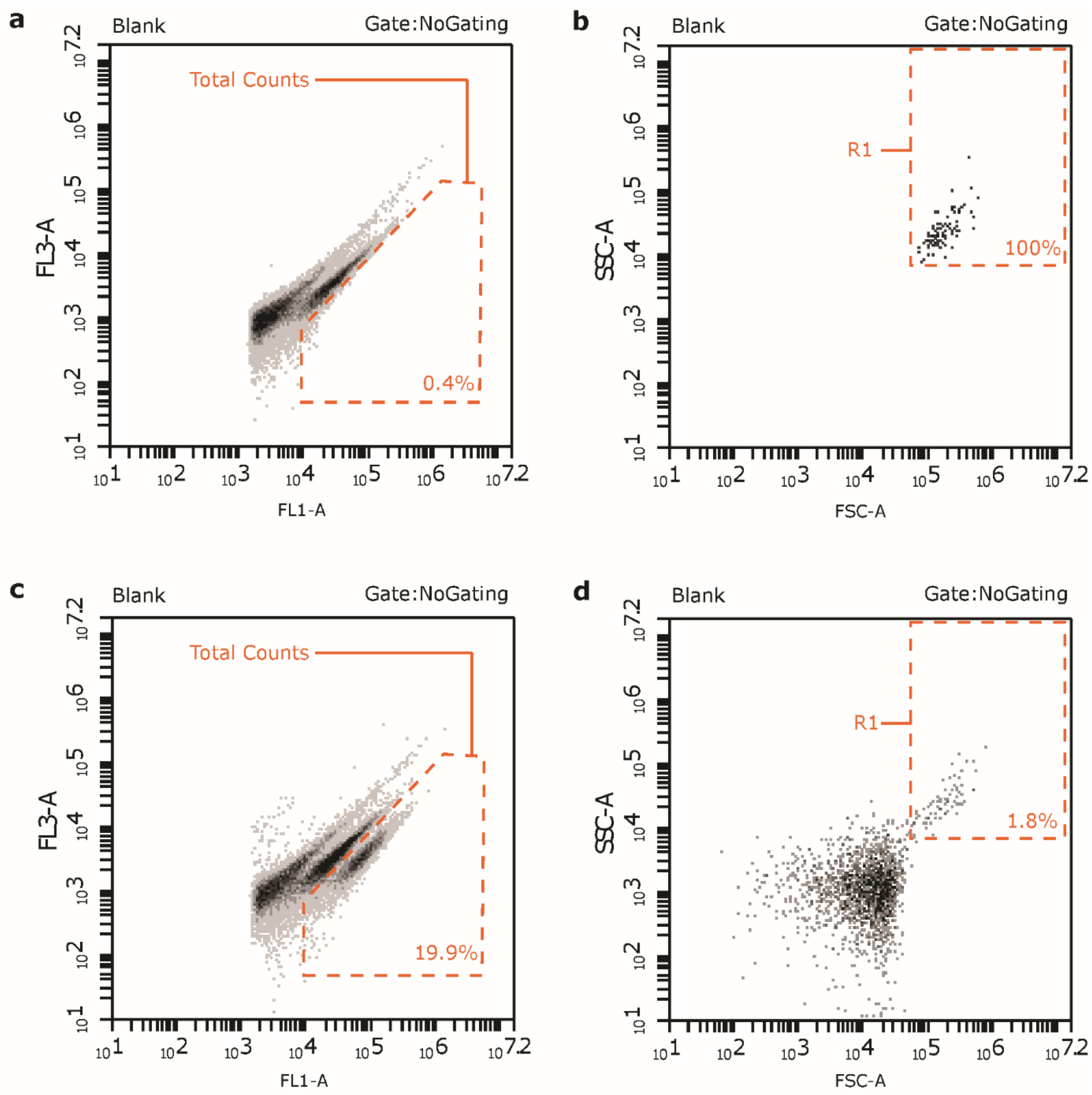

Supplementary Figure 8: Gating Strategy Flow cytometry

A fixed gating and staining approach was applied. Both blank and sample solutions were stained with SYBR Green I. **a**, The FL1-A/FL3-A acquisition plot of a blank sample (0.85% w/v physiological solution) with gate boundaries indicated. A threshold value of 2,000 was applied on the FL1 channel. **b**, Secondary gating was performed on the FSC-A/SSC-A channels to further discriminate between debris or background and microbial events. **c**, **d**, FL1-A/FL3-A count acquisition of a faecal sample (**c**) with secondary gating on FSC-A/SSC-A channels on the basis of blank analyses (**d**). Total counts were defined as events registered in the FL1-A/FL3-A gating area, excluding debris or background events observed in the FSC-A/SSC-A R1 gate. The flow rate was set at 14 µl per minute and the acquisition rate did not exceed 10,000 events per second. Each panel reflects events registered over the course of a 30-s acquisition period.

Supplementary Table 1: Determination of lag-time. Proposed lag time based on cross-correlation analysis between stool moisture and the P/C ratio of the previous day for each participant, slope and significance of the correlation, related information, and final lag time as used in the analyses.

Participant	Proposed lag time *	Associated slope	Significant?	Median stool moisture (%)	Median time since last defecation (h)	Time of sampling	Final lag time
800	-4	-0.263856122	yes	73.09	30	Afternoon:12_Evening:6_Morning:10_Night:1	-4
801	-1	-0.302007752	yes	78.07	24	Morning:42	-1
802	-2	-0.166510592	no	70.98	23.75	Afternoon:11_Evening:5_Morning:14	-1
803	-3	-0.067174532	no	75.9	14.5	Afternoon:4_Morning:41	-1
804	-1	-0.170934213	no	72.53	32.5	Afternoon:5_Evening:5_Morning:11	-1
805	-2	-0.408542242	yes	82.71	22.5	Afternoon:3_Evening:1_Morning:35	-2
806	-2	-0.189618863	no	77.07	22.5	Afternoon:8_Evening:9_Morning:8_Night:1	-1
807	-1	-0.195957759	no	60.23	17.5	Afternoon:1_Morning:39	-1
808	-4	-0.091100488	no	70.805	25	Afternoon:11_Evening:11_Morning:10_Night:1	-1
809	-2	-0.226582804	no	74.84	23.5	Afternoon:10_Evening:13_Morning:7_Night:1	-1
810	-5	-0.343098783	yes	67.01	22.5	Afternoon:5_Evening:19_Morning:14	-5
811	-5	-0.19020666	no	75.94	14	Afternoon:7_Evening:5_Morning:31_Night:1	-1
814	-2	-0.147461484	no	67.51	16	Afternoon:5_Evening:2_Morning:22_Night:1	-1
815	0	-0.510107276	yes	77.98	25	Afternoon:4_Evening:13_Morning:14	0
816	-2	-0.029807728	no	86.18	14	Afternoon:22_Evening:1_Morning:14	-1
817	0	-0.183358611	no	67.94	24.5	Afternoon:1_Morning:31	-1
818	-2	-0.288589038	no	70.155	24	Afternoon:1_Evening:3_Morning:37	-1
819	-3	-0.207215476	no	81.18	23	Afternoon:2_Evening:1_Morning:36	-1
820	-1	-0.249295696	yes	68.57	14	Afternoon:8_Evening:10_Morning:29_Night:1	-1
821	-2	0.037792959	no	74.12	12	Morning:45	-1

Supplementary Table 2: Taylor law relationschips per genus. For each genus, the linearity of the relationship between log(variance) and log(mean) over time was tested using function lm, calculating slopes, p-values and adjusted R^2.

	slope	pvalue	qvalue	adjR2	excluded
g_Acetanaerobacterium	NA	NA	NA	NA	Yes, < abundance threshold 0.5%
g_Acetitomaculum	NA	NA	NA	NA	Yes, < abundance threshold 0.5%
g_Acidaminococcus	NA	NA	NA	NA	Yes, < abundance threshold 0.5%
g_Actinomyces	NA	NA	NA	NA	Yes, < abundance threshold 0.5%
g_Adlercreutzia	1.318982	4.32E-10	8.53E-10	0.925856	No
g_Aestuariispira	1.719915	6.36E-08	8.59E-08	0.930594	No
g_Akkermansia	1.808826	4.02E-09	6.86E-09	0.915801	No
g_Alistipes	1.719098	1.67E-11	5.80E-11	0.919539	No
g_Allisonella	NA	NA	NA	NA	Yes, < abundance threshold 0.5%
g_Allobaculum	NA	NA	NA	NA	Yes, present in < 5 ind with > 3 timepoints per timeseries
g_Alloprevotella	NA	NA	NA	NA	Yes, present in < 5 ind with > 3 timepoints per timeseries
g_Anaerofilum	NA	NA	NA	NA	Yes, < abundance threshold 0.5%
g_Anaerofustis	NA	NA	NA	NA	Yes, < abundance threshold 0.5%
g_Anaerorhabdus	NA	NA	NA	NA	Yes, < abundance threshold 0.5%
g_Anaerosporobacter	1.272728	4.39E-12	1.89E-11	0.968111	No
g_Anaerostipes	1.926801	4.15E-09	6.89E-09	0.852016	No
g_Anaerotruncus	1.177491	0	0	0.988168	No
g_Arcanobacterium	NA	NA	NA	NA	Yes, < abundance threshold 0.5%
g_Bacteroides	1.897164	3.68E-10	7.46E-10	0.886742	No
g_Barnesiella	1.773776	6.85E-09	1.02E-08	0.90917	No
g_Bifidobacterium	1.744071	8.44E-15	2.05E-13	0.977337	No
g_Bilophila	1.585377	2.79E-10	5.98E-10	0.930058	No
g_Blautia	2.006126	7.69E-11	2.21E-10	0.904727	No
g_Butyricicoccus	1.628223	4.58E-09	7.28E-09	0.850388	No
g_Butyricimonas	1.806271	6.80E-09	1.02E-08	0.893146	No
g_Butyrivibrio	NA	NA	NA	NA	Yes, present in < 5 ind with > 3 timepoints per timeseries
g_Campylobacter	NA	NA	NA	NA	Yes, < abundance threshold 0.5%
g_Catabacter	NA	NA	NA	NA	Yes, < abundance threshold 0.5%
g_Catenibacterium	NA	NA	NA	NA	Yes, present in < 5 ind with > 3 timepoints per timeseries
g_Cerasicoccus	1.470821	6.10E-07	7.68E-07	0.937922	No
g_Christensenella	NA	NA	NA	NA	Yes, < abundance threshold 0.5%
g_Citrobacter	NA	NA	NA	NA	Yes, < abundance threshold 0.5%
g_Cloacibacillus	NA	NA	NA	NA	Yes, < abundance threshold 0.5%
g_Clostridium_III	NA	NA	NA	NA	Yes, < abundance threshold 0.5%
g_Clostridium_IV	1.773913	7.11E-15	2.05E-13	0.96593	No
g_Clostridium_sensu_stricto	NA	NA	NA	NA	Yes, < abundance threshold 0.5%
g_Clostridium_XlVa	1.941787	4.17E-07	5.34E-07	0.754381	No
g_Clostridium_XlVb	1.440485	7.76E-13	4.72E-12	0.951729	No
g_Clostridium_XVIII	1.778503	1.15E-10	2.89E-10	0.900429	No
g_Collinsella	2.038511	7.35E-14	1.07E-12	0.963396	No
g_Comamonas	NA	NA	NA	NA	Yes, < abundance threshold 0.5%
g_Coprobacillus	NA	NA	NA	NA	Yes, < abundance threshold 0.5%

g_Coprobacter	1.408486	3.03E-09	5.40E-09	0.903993	No
g_Coprococcus	1.817	3.13E-12	1.43E-11	0.952627	No
g_Corynebacterium	NA	NA	NA	NA	Yes, < abundance threshold 0.5%
g_Defluviitalea	NA	NA	NA	NA	Yes, < abundance threshold 0.5%
g_Desulfomicrobium	NA	NA	NA	NA	Yes, present in < 5 ind with > 3 timepoints per timeseries
g_Desulfovibrio	1.379804	3.69E-05	3.85E-05	0.943116	No
g_Dialister	1.740359	3.65E-05	3.85E-05	0.847092	No
g_Dorea	1.592714	4.34E-11	1.38E-10	0.910568	No
g_Eggerthella	NA	NA	NA	NA	Yes, < abundance threshold 0.5%
g_Eggerthia	NA	NA	NA	NA	Yes, present in < 5 ind with > 3 timepoints per timeseries
g_Eisenbergiella	NA	NA	NA	NA	Yes, < abundance threshold 0.5%
g_Elusimicrobium	NA	NA	NA	NA	Yes, present in < 5 ind with > 3 timepoints per timeseries
g_Enterobacter	NA	NA	NA	NA	Yes, < abundance threshold 0.5%
g_Enterococcus	NA	NA	NA	NA	Yes, present in < 5 ind with > 3 timepoints per timeseries
g_Enterorhabdus	1.529756	0.002209	0.002209	0.960201	No
g_Escherichia.Shigella	1.923327	4.32E-09	7.01E-09	0.867438	No
g_Ethanoligenens	NA	NA	NA	NA	Yes, < abundance threshold 0.5%
g_Eubacterium	NA	NA	NA	NA	Yes, < abundance threshold 0.5%
g_Ezakiella	NA	NA	NA	NA	Yes, < abundance threshold 0.5%
g_Faecalibacterium	1.586017	3.70E-08	5.10E-08	0.8117	No
g_Faecalicoccus	1.292338	3.65E-10	7.46E-10	0.952028	No
g_Faecalitalea	NA	NA	NA	NA	Yes, present in < 5 ind with > 3 timepoints per timeseries
g_Flavonifractor	1.374816	6.42E-10	1.20E-09	0.908063	No
g_Frondihabitans	NA	NA	NA	NA	Yes, < abundance threshold 0.5%
g_Fusicatenibacter	1.687615	1.57E-11	5.73E-11	0.920081	No
g_Fusobacterium	NA	NA	NA	NA	Yes, < abundance threshold 0.5%
g_Gemella	NA	NA	NA	NA	Yes, < abundance threshold 0.5%
g_Gemmiger	1.828643	6.36E-09	9.87E-09	0.861329	No
g_Gordonibacter	1.36327	1.46E-06	1.75E-06	0.980529	No
g_Gracilibacter	NA	NA	NA	NA	Yes, < abundance threshold 0.5%
g_Guggenheimella	NA	NA	NA	NA	Yes, < abundance threshold 0.5%
g_Haemophilus	1.400717	2.44E-10	5.56E-10	0.918503	No
g_Hafnia	NA	NA	NA	NA	Yes, < abundance threshold 0.5%
g_Hespellia	1.604246	2.83E-08	3.98E-08	0.834914	No
g_Holdemanella	NA	NA	NA	NA	Yes, present in < 5 ind with > 3 timepoints per timeseries
g_Holdemania	NA	NA	NA	NA	Yes, < abundance threshold 0.5%
g_Howardella	NA	NA	NA	NA	Yes, < abundance threshold 0.5%
g_Hungatella	NA	NA	NA	NA	Yes, < abundance threshold 0.5%
g_Hydrogenoanaerobacterium	1.34709	7.88E-11	2.21E-10	0.971302	No
g_Intestinibacter	1.205145	9.25E-11	2.50E-10	0.915446	No
g_Intestinimonas	1.522959	7.51E-07	9.30E-07	0.77939	No
g_Lactobacillus	NA	NA	NA	NA	Yes, present in < 5 ind with > 3 timepoints per timeseries
g_Lactococcus	NA	NA	NA	NA	Yes, < abundance threshold 0.5%
g_Lactonifactor	NA	NA	NA	NA	Yes, < abundance threshold 0.5%
g_Lautropia	NA	NA	NA	NA	Yes, < abundance threshold 0.5%
g_Leuconostoc	NA	NA	NA	NA	Yes, < abundance threshold 0.5%

g_Macellibacteroides	NA	NA	NA	NA	Yes, < abundance threshold 0.5%
g_Mannheimia	NA	NA	NA	NA	Yes, < abundance threshold 0.5%
g_Methanobrevibacter	2.221264	9.70E-05	9.97E-05	0.810709	No
g_Methanomassiliicoccus	NA	NA	NA	NA	Yes, present in < 5 ind with > 3 timepoints per timeseries
g_Methanosphaera	NA	NA	NA	NA	Yes, < abundance threshold 0.5%
g_Mitsuokella	NA	NA	NA	NA	Yes, < abundance threshold 0.5%
g_Mogibacterium	NA	NA	NA	NA	Yes, < abundance threshold 0.5%
g_Murdochiella	NA	NA	NA	NA	Yes, < abundance threshold 0.5%
g_Neisseria	NA	NA	NA	NA	Yes, < abundance threshold 0.5%
g_Odoribacter	1.526895	2.22E-06	2.57E-06	0.770637	No
g_Oligosphaera	NA	NA	NA	NA	Yes, present in < 5 ind with > 3 timepoints per timeseries
g_Olsenella	1.708358	0.000836	0.000847	0.941462	No
g_Oscillibacter	1.725078	1.93E-14	3.53E-13	0.961946	No
g_Oxalobacter	NA	NA	NA	NA	Yes, < abundance threshold 0.5%
g_Papillibacter	NA	NA	NA	NA	Yes, < abundance threshold 0.5%
g_Parabacteroides	2.365413	3.74E-11	1.24E-10	0.923954	No
g_Paraprevotella	2.391589	1.74E-05	1.92E-05	0.929521	No
g_Parasutterella	1.430587	4.76E-13	3.52E-12	0.976771	No
g_Parvibacter	NA	NA	NA	NA	Yes, < abundance threshold 0.5%
g_Parvimonas	NA	NA	NA	NA	Yes, < abundance threshold 0.5%
g_Pelospora	NA	NA	NA	NA	Yes, < abundance threshold 0.5%
g_Peptococcus	NA	NA	NA	NA	Yes, < abundance threshold 0.5%
g_Peptoniphilus	NA	NA	NA	NA	Yes, < abundance threshold 0.5%
g_Phascolarctobacterium	1.871308	1.51E-05	1.70E-05	0.903855	No
g_Porphyromonas	NA	NA	NA	NA	Yes, < abundance threshold 0.5%
g_Prevotella	1.668891	2.02E-10	4.92E-10	0.989488	No
g_Proteus	NA	NA	NA	NA	Yes, < abundance threshold 0.5%
g_Pseudobutyrivibrio	NA	NA	NA	NA	Yes, < abundance threshold 0.5%
g_Pseudoflavonifractor	1.327879	5.36E-13	3.56E-12	0.953775	No
g_Raoultella	NA	NA	NA	NA	Yes, < abundance threshold 0.5%
g_Rikenella	NA	NA	NA	NA	Yes, present in < 5 ind with > 3 timepoints per timeseries
g_Romboutsia	1.278416	1.11E-10	2.89E-10	0.913648	No
g_Roseburia	1.652482	5.24E-10	1.01E-09	0.882216	No
g_Rothia	NA	NA	NA	NA	Yes, < abundance threshold 0.5%
g_Ruminococcus	1.813903	5.84E-11	1.78E-10	0.919877	No
g_Ruminococcus2	1.729004	1.46E-11	5.62E-11	0.920702	No
g_Senegalimassilia	1.523291	2.24E-08	3.27E-08	0.981002	No
g_Slackia	1.405686	4.04E-09	6.86E-09	0.969562	No
g_Solobacterium	NA	NA	NA	NA	Yes, < abundance threshold 0.5%
g_Sporobacter	1.576867	2.22E-10	5.24E-10	0.944212	No
g_Staphylococcus	NA	NA	NA	NA	Yes, < abundance threshold 0.5%
g_Streptococcus	1.635934	1.24E-11	5.04E-11	0.922133	No
g_Subdoligranulum	1.451881	1.82E-12	9.49E-12	0.946651	No
g_Succinatimonas	NA	NA	NA	NA	Yes, < abundance threshold 0.5%
g_Succiniclasticum	NA	NA	NA	NA	Yes, < abundance threshold 0.5%
g_Sutterella	1.920742	8.70E-08	1.15E-07	0.88912	No

g_Terrisporobacter	NA	NA	NA	NA	Yes, < abundance threshold 0.5%
g_Turicibacter	NA	NA	NA	NA	Yes, < abundance threshold 0.5%
g_Ureaplasma	NA	NA	NA	NA	Yes, < abundance threshold 0.5%
g_Vampirovibrio	NA	NA	NA	NA	Yes, present in < 5 ind with > 3 timepoints per timeseries
g_Varibaculum	NA	NA	NA	NA	Yes, < abundance threshold 0.5%
g_Veillonella	1.426921	1.19E-12	6.71E-12	0.980074	No
g_Victivallis	1.580487	1.97E-06	2.32E-06	0.895632	No
g_Weissella	NA	NA	NA	NA	Yes, < abundance threshold 0.5%
g_Yersinia	NA	NA	NA	NA	Yes, < abundance threshold 0.5%
uc_c_Alphaproteobacteria	NA	NA	NA	NA	Yes, present in < 5 ind with > 3 timepoints per timeseries
uc_c_Clostridia	1.227716	3.21E-13	2.96E-12	0.983721	No
uc_c_Deltaproteobacteria	NA	NA	NA	NA	Yes, present in < 5 ind with > 3 timepoints per timeseries
uc_c_Gammaproteobacteria	NA	NA	NA	NA	Yes, < abundance threshold 0.5%
uc_c_Opitutae	NA	NA	NA	NA	Yes, < abundance threshold 0.5%
uc_f_Clostridiales_Incertae_Sedis_XIII	NA	NA	NA	NA	Yes, < abundance threshold 0.5%
uc_f_Coriobacteriaceae	1.715084	4.82E-13	3.52E-12	0.962483	No
uc_f_Cryomorphaceae	NA	NA	NA	NA	Yes, < abundance threshold 0.5%
uc_f_Desulfovibrionaceae	NA	NA	NA	NA	Yes, present in < 5 ind with > 3 timepoints per timeseries
uc_f_Enterobacteriaceae	NA	NA	NA	NA	Yes, present in < 5 ind with > 3 timepoints per timeseries
uc_f_Erysipelotrichaceae	1.423754	2.60E-08	3.73E-08	0.940943	No
uc_f_Lachnospiraceae	1.743849	1.42E-07	1.86E-07	0.781691	No
uc_f_Peptostreptococcaceae	NA	NA	NA	NA	Yes, < abundance threshold 0.5%
uc_f_Porphyromonadaceae	1.488836	1.38E-06	1.68E-06	0.92567	No
uc_f_Prevotellaceae	NA	NA	NA	NA	Yes, present in < 5 ind with > 3 timepoints per timeseries
uc_f_Rhodospirillaceae	1.741228	6.81E-10	1.24E-09	0.986237	No
uc_f_Rikenellaceae	1.423355	2.42E-05	2.64E-05	0.973752	No
uc_f_Ruminococcaceae	1.747086	2.64E-13	2.96E-12	0.949162	No
uc_f_Sutterellaceae	NA	NA	NA	NA	Yes, present in < 5 ind with > 3 timepoints per timeseries
uc_f_Synergistaceae	NA	NA	NA	NA	Yes, < abundance threshold 0.5%
uc_f_Veillonellaceae	NA	NA	NA	NA	Yes, < abundance threshold 0.5%
uc_k_Bacteria	1.582117	3.24E-13	2.96E-12	0.964295	No
uc_o_Bacillales	NA	NA	NA	NA	Yes, < abundance threshold 0.5%
uc_o_Bacteroidales	NA	NA	NA	NA	Yes, < abundance threshold 0.5%
uc_o_Burkholderiales	NA	NA	NA	NA	Yes, present in < 5 ind with > 3 timepoints per timeseries
uc_o_Clostridiales	1.650315	2.64E-10	5.84E-10	0.904405	No
uc_o_Desulfovibrionales	NA	NA	NA	NA	Yes, < abundance threshold 0.5%
uc_o_Lactobacillales	NA	NA	NA	NA	Yes, < abundance threshold 0.5%
uc_o_Rhodospirillales	NA	NA	NA	NA	Yes, present in < 5 ind with > 3 timepoints per timeseries
uc_p_Bacteroidetes	1.306841	3.07E-05	3.30E-05	0.971147	No
uc_p_Firmicutes	1.581177	2.95E-12	1.43E-11	0.969873	No
uc_p_Lentisphaerae	NA	NA	NA	NA	Yes, < abundance threshold 0.5%
uc_p_Proteobacteria	1.58872	2.81E-06	3.21E-06	0.975789	No
