# Supplementary material for: Temporal variability in quantitative human gut microbiome profiles and implications for clinical research
Source: Nat Commun. 2021 Nov 18;12:6740. doi: 10.1038/s41467-021-27098-7 (PMC8602282; doi:10.1038/s41467-021-27098-7)
Supplement: Supplementary file 2 — Description of Additional Supplementary Files [file 41467_2021_27098_MOESM2_ESM.docx]

**Description of Additional Supplementary Files**

SupplementaryDataS1.xlsx

This file contains the summary statistics of the metadata, the QMP, RMP and non-rarefied profiles of the study samples, all derived microbiome data, the taxonomic table and additional information on the positive and negative controls.

SupplementaryDataS2.xlsx

This file contains data regarding the variation in metadata, taxa abundance, and alpha diversity measurements, and fold-changes in genus abundance.

SupplementaryDataS3.xlsx

This file holds all data on core genera.

SupplementaryDataS4.xlsx

This file contains all tables on Bray Curtis Dissimilarity between samples of the study cohort, as well as the results of related statistical tests.

SupplementaryDataS5.xlsx

This file contains all results of statistical tests regarding the relations between taxa abundance/variation and metadata levels/variation.

SupplementaryDataS6.xlsx

Pairwise dissimilarity data used for mislabeling analysis.
