# Supplementary material for: Temporal variability in quantitative human gut microbiome profiles and implications for clinical research
Source: Nat Commun. 2021 Nov 18;12:6740. doi: 10.1038/s41467-021-27098-7 (PMC8602282; doi:10.1038/s41467-021-27098-7)
Supplement: Supplementary file 9 — Reporting Summary [file 41467_2021_27098_MOESM9_ESM.pdf]

nature research

Corresponding author(s): Jeroen Raes

Last updated by author(s): October 18, 2021

Reporting Summary

Nature Research wishes to improve the reproducibility of the work that we publish. This form provides structure for consistency and transparency in reporting. For further information on Nature Research policies, see Authors & Referees and the Editorial Policy Checklist .

Statistics

For all statistical analyses, confirm that the following items are present in the figure legend, table legend, main text, or Methods section.

n/a	Confirmed	
☐	☒	The exact sample size (*n*) for each experimental group/condition, given as a discrete number and unit of measurement
☐	☒	A statement on whether measurements were taken from distinct samples or whether the same sample was measured repeatedly
☐	☒	The statistical test(s) used AND whether they are one- or two-sided<br>*Only common tests should be described solely by name; describe more complex techniques in the Methods section.*
☐	☒	A description of all covariates tested
☐	☒	A description of any assumptions or corrections, such as tests of normality and adjustment for multiple comparisons
☐	☒	A full description of the statistical parameters including central tendency (e.g. means) or other basic estimates (e.g. regression coefficient) AND variation (e.g. standard deviation) or associated estimates of uncertainty (e.g. confidence intervals)
☐	☒	For null hypothesis testing, the test statistic (e.g. *F*, *t*, *r*) with confidence intervals, effect sizes, degrees of freedom and *P* value noted<br>*Give P values as exact values whenever suitable.*
☒	☐	For Bayesian analysis, information on the choice of priors and Markov chain Monte Carlo settings
☒	☐	For hierarchical and complex designs, identification of the appropriate level for tests and full reporting of outcomes
☐	☒	Estimates of effect sizes (e.g. Cohen's *d*, Pearson's *r*), indicating how they were calculated

Our web collection on statistics for biologists contains articles on many of the points above.

Software and code

Policy information about availability of computer code

Data collection	An open source QMP R-script is available in the Github repository (https://github.com/raeslab/QMP) with public access.<br>Raw sequencing data were deposited in the European Genome Archive (EGA) with accession number:
Data analysis	Data analysis were performed in the open source software R.<br>Flow cytometry measurements made use of the BD Accuri CFlow software.

For manuscripts utilizing custom algorithms or software that are central to the research but not yet described in published literature, software must be made available to editors/reviewers. We strongly encourage code deposition in a community repository (e.g. GitHub). See the Nature Research guidelines for submitting code & software for further information.

Data

Policy information about availability of data

All manuscripts must include a data availability statement. This statement should provide the following information, where applicable:

- Accession codes, unique identifiers, or web links for publicly available datasets
- A list of figures that have associated raw data
- A description of any restrictions on data availability

Supplementary Table S1 contains the QMP, RMP and non-rarefied profiles of the samples and controls, the taxonomic table, and the microbiome derived data. Raw amplicon sequencing data that support the findings of this study have been deposited in the European Genome-Phenome Archive with accession code XX (http://www.ebi.ac.uk/ena/data/view/XX) with public access. Source data for all figures are provided with the paper. Additional data requests can be directed to the corresponding author.

Field-specific reporting

Please select the one below that is the best fit for your research. If you are not sure, read the appropriate sections before making your selection.

☒ Life sciences ☐ Behavioural & social sciences ☐ Ecological, evolutionary & environmental sciences

For a reference copy of the document with all sections, see nature.com/documents/nr-reporting-summary-flat.pdf

Life sciences study design

All studies must disclose on these points even when the disclosure is negative.

Sample size	At the moment of study set up, there were no available methods for sample size estimation and little information on effect size in microbiome studies. We here aimed for a sample size of 20 individuals. Sample size estimation was based on a case study showing a significant shift of some gut bacteria during the menstruation phase and a longitudinal diet intervention study showing that a significant effect of diet could be noted with daily sampling in 10 individuals. Previously, we were able to show a significant effect of stool consistency with 53 independent datapoints. The longitudinal set up of this study would lead to replicated data, increasing the power of the analysis by incorporating the temporal variation, hence allowing a smaller sample size. However, sample sizes of non-parametric tests for testing differences between two or more groups should generally be >10 to be valuable. We therefore opted for a sample size of 20. The study design, which included one and a half menstrual cycle, allowed to repeat measurements for several menstrual phase parameters, increasing the power of those analyses further.
Data exclusions	Of twenty-two recruited volunteers, two did not complete the study protocol and were excluded from analyses.<br>For statements regarding normal temporal variation, only non-perturbed time-series were included, leaving out one time-series in which an infection event took place.
Replication	This study includes a discovery cohort only. Replication was not performed.
Randomization	This study did not allocate participants into groups, hence no randomization was applied. Factors known to influence microbiome variation (e.g. BMI, age, stool consistency, dietary information, ...) were recorded, summary measures were determined and outliers were investigated to evaluate possible confounding. Statistical analyses were performed considering confounding factors and linear relationships between the collected data.
Blinding	This study did not involve allocation of participants into groups.

Reporting for specific materials, systems and methods

We require information from authors about some types of materials, experimental systems and methods used in many studies. Here, indicate whether each material, system or method listed is relevant to your study. If you are not sure if a list item applies to your research, read the appropriate section before selecting a response.

Materials & experimental systems

n/a	Involved in the study	
☒	☐	Antibodies
☒	☐	Eukaryotic cell lines
☒	☐	Palaeontology
☒	☐	Animals and other organisms
☐	☒	Human research participants
☒	☐	Clinical data

Methods

n/a	Involved in the study	
☒	☐	ChIP-seq
☐	☒	Flow cytometry
☒	☐	MRI-based neuroimaging

Human research participants

Policy information about studies involving human research participants

Population characteristics	Women were eligible to participate if they were aged between 16 and 55 years. Exclusion criteria were the use of any type of hormonal contraception three months prior to or during the study, the use of a copper intrauterine device, antibiotic treatment three months prior to study onset, pregnancy, the presence of inflammatory bowel disease or any type of bowel cancer. In order to be able to assess a possible effect of the menstrual cycle as well as the sex-associated differences in stool consistency, an important parameter for microbiota composition, we only included women in this study.
Recruitment	Participants were recruited in the Flemish region near the university hospital (Leuven, Belgium) through a newsletter directed at FGFP participants as well as flyers distributed throughout the hospital. Volunteers got the provided smart phone as compensation for participation after completion of the study. Selection bias could have been induced through the recruitment channels (people interested in gut microbiome research tend to have gut problems or be related to people with gastrointestinal diseases), strict exclusion criteria, and smart phone use.
Ethics oversight	All experimental protocols were approved by the Commissie Medische Ethiek, UZ KU Leuven. Ethical approval of the study protocol was obtained (B322201525874). Study design complied with all relevant ethical regulations, aligning with the Declaration of Helsinki and in accordance with Belgian privacy.

Note that full information on the approval of the study protocol must also be provided in the manuscript.

Flow Cytometry

Plots

Confirm that:

- [x] The axis labels state the marker and fluorochrome used (e.g. CD4-FITC).
- [x] The axis scales are clearly visible. Include numbers along axes only for bottom left plot of group (a 'group' is an analysis of identical markers).
- [x] All plots are contour plots with outliers or pseudocolor plots.
- [x] A numerical value for number of cells or percentage (with statistics) is provided.

Methodology

Sample preparation	For cell counting, 0.2g of frozen fecal aliquots were diluted 100,000 times in physiological solution (8.5g/l NaCl; VWR International). In order to remove debris from the faecal solutions, samples were filtered using a sterile syringe filter (pore size 5μm; Sartorius Stedim Biotech GmbH). Next, 1ml of the microbial cell suspension obtained was stained with 1μl SYBR Green I (1:100 dilution in dimethylsulfoxide; shaded 15 min incubation at 37°C; 10,000 concentrate, Thermo Fisher Scientific).
Instrument	The flow cytometry analysis of the microbial cells present in the suspension was performed using a C6 Accuri flow cytometer (BD Biosciences), according to previously published methods7.
Software	Fluorescence events were monitored using the FL1 533/30 nm and FL3>670 nm optical detectors. Forward and sideways-scattered light was also collected. The BD Accuri CFlow software was used to gate and separate the microbial fluorescence events on the FL1–FL3 density plot from the faecal sample background.
Cell population abundance	The gated fluorescence events were evaluated on the forward–sideways density plot, to exclude remaining background events and to obtain an accurate microbial cell count.
Gating strategy	Instrument and gating settings were identical for all samples (fixed staining–gating strategy).

- [x] Tick this box to confirm that a figure exemplifying the gating strategy is provided in the Supplementary Information.
